# A role for the transcription factor Mca1 in activating the meiosis-specific copper transporter Mfc1

**DOI:** 10.1371/journal.pone.0201861

**Published:** 2018-08-07

**Authors:** Jude Beaudoin, Raphaël Ioannoni, Vincent Normant, Simon Labbé

**Affiliations:** Département de Biochimie, Faculté de médecine et des sciences de la santé, Université de Sherbrooke, Sherbrooke, QC, Canada; Auburn University, UNITED STATES

## Abstract

When reproduction in fungi takes place by sexual means, meiosis enables the formation of haploid spores from diploid precursor cells. Copper is required for completion of meiosis in *Schizosaccharomyces pombe*. During the meiotic program, genes encoding copper transporters exhibit distinct temporal expression profiles. In the case of the major facilitator copper transporter 1 (Mfc1), its maximal expression is induced during middle-phase meiosis and requires the presence of the Zn_6_Cys_2_ binuclear cluster-type transcription factor Mca1. In this study, we further characterize the mechanism by which Mca1 affects the copper-starvation-induced expression of *mfc1*^*+*^. Using a chromatin immunoprecipitation (ChIP) approach, results showed that a functional Mca1-TAP occupies the *mfc1*^*+*^ promoter irrespective of whether this gene is transcriptionally active. Under conditions of copper starvation, results showed that the presence of Mca1 promotes RNA polymerase II (Pol II) occupancy along the *mfc1*^*+*^ transcribed region. In contrast, Pol II did not significantly occupy the *mfc1*^*+*^ locus in meiotic cells that were incubated in the presence of copper. Further analysis by ChIP assays revealed that binding of Pol II to chromatin at the chromosomal locus of *mfc1*^*+*^ is exclusively detected during meiosis and absent in cells proliferating in mitosis. Protein function analysis of a series of internal mutants compared to the full-length Mca1 identified a minimal form of Mca1 consisting of its DNA-binding domain (residues 1 to 150) fused to the amino acids 299 to 600. This shorter form is sufficient to enhance Pol II occupancy at the *mfc1*^*+*^ locus under low copper conditions. Taken together, these results revealed novel characteristics of Mca1 and identified an internal region of Mca1 that is required to promote Pol II-dependent *mfc1*^*+*^ transcription during meiosis.

## Introduction

Meiosis is a specialized type of cell division through which sexually reproducing organisms produce haploid gametes from diploid precursor cells [1–3]. In the model organism *Schizosaccharomyces pombe*, a diploid precursor cell is formed by conjugation of two haploid cells of opposite mating types. Following conjugation, the two haploid nuclei fuse and that subsequently induces replication of chromosomal DNA, generating pairs of homologous chromosomes. After meiotic recombination between homologous chromosomes that increases genetic diversity, two successive meiotic divisions occur in which homologous chromosomes (meiosis I) and then sister chromatids (meiosis II) are segregated without any intervening DNA replication phase. These two divisions are followed by a differentiation program that includes forespore membrane biogenesis and production of four mature haploid spores (gametes) that are enclosed in an ascus [4, 5].

A number of studies have shown that transition metals such as copper, iron, and zinc are essential for meiosis [6–10]. In murine oocytes, zinc insufficiency blocks the exit from meiosis I [10]. In the case of female porcine gametes, zinc-insufficient oocytes fail to segregate homologous chromosomes, exhibiting an arrest at metaphase I [11]. Similarly, studies with the yeast *S*. *pombe* have shown that cells undergo a meiotic block at metaphase I when copper or iron concentrations are insufficient [6, 7]. In order to identify proteins involved in the mechanisms by which transition metal insufficiency perturbs the meiotic program, studies have been undertaken using different model systems. Among them, *S*. *pombe* is of special interest as growth conditions and temperature-sensitive mutants have been developed that allow synchronization of zygotic cells prior their entry into the meiotic program [12]. For example, a strain harboring a *pat1-114* mutation produces a temperature-sensitive Pat1^114^ kinase. Under low ambient temperature, Pat1^114^ is active and inhibits cells from entering meiosis. In *pat1-114* cells undergoing a transition from low to elevated temperature, Pat1^114^ is readily inactivated and that triggers a cell cycle switch from mitosis to meiosis in a highly efficient and synchronous manner [13, 14].

Investigation of copper homeostasis proteins during meiosis in *S*. *pombe* has revealed that the copper transporters Ctr4 and Ctr5 co-localize at the plasma membrane of zygotic cells shortly after induction of meiosis under low copper conditions [15]. In the case of Ctr6, which is the third member of the copper transporter (Ctr) family in fission yeast, the protein localizes to vacuolar membranes in early meiosis and then undergoes redistribution in a time-dependent manner to reach forespore membranes where it persists until sporulation [15]. In cells proliferating in mitosis as well as cells entering and progressing throughout the meiotic cell cycle, the copper starvation-dependent transcriptional induction of *ctr4*^*+*^, *ctr5*^*+*^, and *ctr6*^*+*^ genes is under the control of the copper-dependent transcription factor Cuf1 [6, 15]. In the case of *ctr6*^*+*^, its meiosis-specific expression also requires the participation of the meiotic regulator Mei4 [15]. DNA microarrays using probes derived from RNA isolated from copper-starved versus copper-replete meiotic cells allowed the identification of *mfc1*^*+*^ [6]. The *mfc1*^*+*^ transcript is the most highly expressed of all of the mRNAs detected in meiotic cells under copper-limiting conditions [6]. Under the control of its own promoter, *mfc1*^*+*^ expression was exclusively detected during meiosis in copper-starved cells. Mfc1 belongs to the major facilitator superfamily (MFS) of transporters and lacks overall sequence homology with Ctr-like proteins. At the protein level, Mfc1 is produced and observed throughout the meiotic divisions and spore maturation process. Mfc1 localizes in the surroundings of the forespore membrane and its presence is required for copper accumulation into forespores and production of fully active copper amine oxidases [6]. Investigation of the full-scale meiotic copper-dependent transcriptional program has revealed that *mfc1*^*+*^ exhibits a distinct temporal expression profile when compared to that of *ctr4*^*+*^ and *ctr5*^*+*^. Whereas *ctr4*^*+*^ and *ctr5*^*+*^ are primarily expressed within the first 3 h of meiosis, followed by their repression after metaphase I, *mfc1*^*+*^ is transcriptionally induced after the 3-h meiotic time point, with its transcript levels remaining sustained throughout the meiotic program [6, 15]. A second important distinction is that, the deletion of *cuf1∆* impairs the transcriptional induction of *ctr4*^*+*^ and *ctr5*^*+*^ but *mfc1*^*+*^ remains unaffected, suggesting the existence of a distinct transcriptional regulator for induction of *mfc1*^*+*^ in response to Cu starvation [6, 15].

Functional analysis of regions in the promoter of *mfc1*^*+*^ has demonstrated that two TCGGCG *cis*-acting elements harboring CGG triplets are necessary for transcriptional activation of *mfc1*^*+*^ under copper-limiting conditions [16]. Consistent with the fact that several regulatory elements containing CGG triplets are known to serve as binding sites for members of the Zn_2_Cys_6_ cluster transcriptional regulator family, Mca1 (a Zn_2_Cys_6_ transcription factor) is required for maximum copper-starvation-induced expression of *mfc1*^*+*^ [16]. Strikingly, developing zygotic *mca1∆*/*mca1∆* cells incubated in the presence of the copper chelator TTM undergo a meiotic block at metaphase I [16]. With respect to primary features of Mca1, its N-terminal region contains one zinc-finger unit that includes six Cys residues in the pattern CX_2_CX_6_CX_6_CX_2_CX_6_C (residues 24–51) which are known to coordinate two zinc ions [17–19]. The zinc finger unit of Mca1 is followed by a linker region (residues 52–116). Although sequence alignments show an absence of similarities between linkers in various Zn_2_Cys_6_ cluster transcription factors, the linker region generally determines DNA half-site spacing and orientation preference of *cis* elements containing CGG triplets [17, 20]. In the case of Mca1, the fact that its linker region is unusually large may justify its capability to bind the two CCG triplets that are found in the direct palindromic TCGGCGN_13_TCGGCG sequence within the *mfc1*^*+*^ promoter. Within this palindrome (TCGGCGN_13_TCGGCG), the distance between the two CGG triplets is 16 bp, which is an atypical long stretch of nucleotides compared to canonical TCGGCG elements containing CGG trinucleotides. Analysis of the amino acid sequence of Mca1 has identified a region that is composed of three heptad repeats of Leu residues (positions 117 to 138). This region is predicted to form an α-helix and may constitute a dimer-forming region of Mca1 that is involved in an intermolecular Mca1-Mca1 interaction. Alternatively, this region may be involved in the formation of a heterodimeric complex with Mca1 and another member of the Zn_2_Cys_6_ cluster protein family or, a partner from another transcription factor family [17, 20]. Based upon this observation and as reported in the case of several Zn_2_Cys_6_ cluster transcription factors, the zinc finger unit, the linker and the dimerization region would form the DNA-binding domain of Mca1. *In vitro* binding studies have consistently shown that the N-terminal 150 amino acids of Mca1 specifically associates with the palindromic TCGGCGN_13_TCGGCG sequence of the *mfc1*^*+*^ promoter region [16]. A large region that includes three sub-regions which are termed ID1, ID2, and ID3 (inhibitory domains) is located C-terminal to the DNA binding domain of Zn_2_Cys_6_ cluster transcription factors [21, 22]. The ID1/2/3 sub-regions are also called the middle homology region (MHR) and are thought to participate in regulating the transcriptional activity of zinc binuclear cluster proteins [17, 20–22]. Deletions and mutations within the MHR frequently render Zn_2_Cys_6_ transcriptional regulators constitutively active, suggesting a role for MHR in preventing transactivation in the absence of specific stimuli [17, 20]. With respect to transcriptional activation function of zinc binuclear cluster transcription factors, several of them contain an acidic amino acid region at their C termini that acts as transactivation domain.

In this report, we sought to gain insight into the mechanism by which Mca1 operates to activate *mfc1*^*+*^ gene expression. Using a chromatin immunoprecipitation (ChIP) approach, we showed that Mca1 is constitutively bound to *mfc1*^*+*^ promoter. Under conditions of copper starvation, results of ChIP experiments showed that Mca1 operates through a transcriptional mechanism to promote RNA polymerase II (Pol II) occupancy along the *mfc1*^*+*^ transcribed region. In marked contrast, association of Pol II with *mfc1*^*+*^ locus drastically decreases under copper-replete conditions. Further ChIP analysis showed that, although Mca1 occupies the *mfc1*^*+*^ promoter in vegetative cells, Pol II occupancy along the *mfc1*^*+*^ transcribed region is exclusively detected during meiosis. Deletion analysis of the Mca1 protein revealed that a central portion (residues 299 to 600) fused to the DNA-binding domain (residues 1 to 150) of Mca1 was required to enhance Pol II occupancy at the *mfc1*^*+*^ locus in meiotic cells under low copper conditions. Taken together, these findings reveal novel features of Mca1, a key regulator required for promoting Pol II-dependent *mfc1*^*+*^ transcription.

## Materials and methods

### Strains and media

*S*. *pombe* strains undergoing vegetative growth were isogenic derivatives of the parental strain FY435 (*h*^*+*^ *his7-366 leu1-32 ura4-∆18 ade6-M210*) [16] and their genotypes are described as follows: *cuf1∆* (*h*^*+*^ *his7-366 leu1-32 ura4-∆18 ade6-M210 cuf1∆*::*KAN*^*r*^) and *mca1∆* (*h*^*+*^ *his7-366 leu1-32 ura4-∆18 ade6-M210 mca1∆*::*KAN*^*r*^). In the case of strains that entered into meiosis, we used isogenic strains in which the *pat1-114* temperature-sensitive mutation allowed synchronization of cells in terms of their entry into the meiotic program. Genotypes of strains harboring a *pat1-114* mutation are summarized as follows: *h*^*+*^ *pat1-114 ade6-M210* (JB484) [23], *h*^*+*^ *pat1-114 ade6-M216* (JB485) [23], *h*^*+*^ *pat1-114 ade6-M210 mca1∆*::*KAN*^*r*^ (JSY26), *h*^*+*^ *pat1-114 ade6-M216 mca1∆*::*KAN*^*r*^ (JSY27), and *h*^*+*^*/h*^*+*^ *pat1-114/pat1-114 ade6-M210/ade6-M216 mca1∆*::*KAN*^*r*^*/mca1∆*::*KAN*^*r*^ (JSY30). Standard methods were used for growth, sexual conjugation and sporulation of cells [4]. Non-selective yeast extract plus supplements (YES) medium for *S*. *pombe* contained 0.5% yeast extract, 3% glucose, and 225 mg/l of adenine, histidine, leucine, uracil and lysine. Strains used for DNA plasmid integration were grown in Edinburgh minimal medium (EMM), in which specific nutrients were lacking as required to foster chromosomal integration events. *h*^*+*^/*h*^*+*^ *pat1-114/pat1-114* and *h*^*+*^/*h*^*+*^ *pat1-114/pat1-114 mca1∆/mca1∆* diploid strains were generated by incubating mid-logarithmic phase cultures of haploid cells with 20 μg/ml carbendazim (Sigma-Aldrich) as described previously [24]. Preparation and synchronization of *pat1-114*/*pat1-114* diploid cells for their entry into meiosis was performed as described previously [6].

### Plasmids

The *mca1*^*+*^ promoter containing 500 bp of the 5’ noncoding region and the first 150 codons (450 bp) of the *mca1*^*+*^ gene was isolated by PCR, using primers designed to generate ApaI and XmaI restriction sites at the termini of the PCR product. The PCR product obtained was digested with ApaI and XmaI and cloned into the corresponding sites of pBPade6 [25]. The resulting plasmid was designated pBPade6prom-mca1DBD. To create a plasmid containing full-length Mca1, an XmaI-SacII PCR-amplified fragment containing the last 547 codons of the *mca1*^*+*^ ORF was isolated. The XmaI-SacII DNA fragment was inserted in-frame into the corresponding sites of pBPade6prom-mca1DBD, generating a plasmid that was denoted pBPade6prom-*mca1*^*+*^. To construct a TAP epitope-tagged Mca1, the TAP coding sequence derived from Cuf2-TAP [26] was isolated by PCR using primers designed to generate SacII and SacI sites at the 5’ and 3’ termini of the TAP ORF. The resulting DNA fragment was used to clone the TAP coding sequence into pBPade6prom-*mca1*^*+*^ plasmid to which SacII and SacI restriction sites had previously been introduced by PCR and placed immediately before the *mca1*^*+*^ stop codon. For this particular construct, named pBPade6prom-*mca1*^*+*^*-TAP*, the SacII-SacI TAP-encoded fragment was inserted in-frame with the C-terminal region of Mca1.

Two distinct ApaI-SacII PCR-amplified fragments containing the *mca1*^*+*^ locus starting at -500 bp relative to the A of the ATG codon up to +1710 and +1800 bp after the initiator codon were isolated. These two DNA fragments were swapped with a corresponding ApaI-SacII DNA fragment into pBPade6prom-*mca1*^*+*^ and pBPade6prom-*mca1*^*+*^*-TAP* plasmids. The resulting plasmids were named pBPade6prom-*mca1*1-570, pBPade6prom-*mca1*1-600, pBPade6prom-*mca1*1-570-TAP, and pBPade6prom-*mca1*1-600-TAP. Two C-terminal coding regions of Mca1 containing the last 398 (residues 299–697) and 371 (residues 326–697) codons were amplified from pBPade6prom-*mca1*^*+*^ and pBPade6prom-*mca1*^*+*^*-TAP* plasmids using two sets of primers designed to introduce XmaI and SacII or XmaI and SacI at the termini of DNA fragments. Each PCR product was purified, digested with XmaI and SacII or SacI, and then inserted into pBPade6prom-mca1DBD at the XmaI and SacII or XmaI and SacI sites. The resulting plasmids were named pBPade6prom-mca1DBD-299-697, pBPade6prom-mca1DBD-299-697-TAP, pBPade6prom-mca1DBD-326-697, and pBPade6prom-mca1DBD-326-697-TAP. A DNA fragment containing the *mca1*^*+*^ promoter up to -500 bp upstream from the start codon and the first 298 codons of the mca1+ gene was amplified from pBPade6prom-*mca1*^*+*^ using primers designed to introduce ApaI and XmaI at the termini of the DNA fragment. The PCR product was purified, digested with ApaI and XmaI, and used to replace an ApaI-XmaI DNA fragment in pBPade6prom-mca1DBD-326-697 or pBPade6prom-mca1DBD-326-697-TAP. The resulting plasmids were denoted pBPade6prom-mca1-1-298+326–697 and pBPade6prom-mca1-1-298+326-697-TAP. The *mca1*^*+*^ coding region corresponding to amino acid residues 601–697 was isolated by PCR using primers that contained XmaI and SacII or XmaI and SacI at their ends. Subsequently, purified XmaI-SacII and XmaI-SacI DNA fragments were exchanged with the XmaI-SacII and XmaI-SacI DNA regions in plasmids pBPade6prom-mca1-1-298+326–697 and pBPade6prom-mca1-1-298+326-697-TAP to generate pBPade6prom-mca1-1-298+601–697 and pBPade6prom-mca1-1-298+601-697-TAP, respectively.

To create the pSK*cuf1*^*+*^ plasmid, an ApaI-XmaI PCR-amplified DNA segment containing the *cuf1*^*+*^ locus starting at -977 bp from the translational start codon up to the stop codon was inserted into the corresponding sites of pBluescript SK (Stratagene). Subsequently, the *myc*_*12*_ epitope coding region was isolated from p*ctr4*^*+*^*-X-myc*_*12*_ plasmid [27] using XmaI and XbaI and then inserted into the corresponding sites of pSK*cuf1*^*+*^ plasmid, resulting in an in-frame fusion of the *cuf1*^*+*^ gene with twelve copies of the Myc epitope. Once created, the *cuf1*^*+*^*-myc*_*12*_ fusion gene and its promoter region were isolated using ApaI and SacI and inserted into the corresponding sites of pBPade6. The resulting integrative plasmid was designated pBPade6*cuf1*^*+*^*-myc*_*12*_. In the case of the untagged *cuf1*^*+*^ allele, the integrative plasmid pBPade6*cuf1*^*+*^ was constructed by subcloning the ApaI-XmaI fragment from pSK*cuf1*^*+*^ into the corresponding sites of pBPade6.

### RNA isolation and analysis

Total RNA was extracted using a hot phenol method as described previously [28]. Gene expression profiles were analyzed using RNase protection assays as described previously [29]. Plasmids pSK*mfc1*^*+*^ [6], pSK*mca1*^*+*^ [16], pSK*ctr4*^*+*^ [30] and pSK*act1*^*+*^ [31] were used to produce antisense RNA probes that served to determine *mfc1*^*+*^, *mca1*^*+*^, *ctr4*^*+*^, and *act1*^*+*^ steady-state mRNA levels, respectively. ^32^P-labeled antisense RNA probes were produced using the above-mentioned BamHI-linearized plasmids and with the use of [α-^32^P]UTP and T7 RNA polymerase. The *act1*^*+*^ riboprobe was used to detect *act1*^*+*^ transcript as an internal control for normalization during quantification of the RNase protection products.

### Protein extraction and analysis

To determine the steady-state protein levels of Mca1-TAP or its mutant derivatives and α-tubulin, whole cell extracts were prepared using a trichloroacetic acid (TCA) extraction method [32]. Equal amounts of each sample preparation were resuspended in sodium dodecyl sulfate loading buffer and proteins were resolved by electrophoresis on 8% sodium dodecyl sulfate-polyacrylamide gels. Proteins were then electroblotted onto nitrocellulose membranes for 1 h at 4°C. Membranes were blocked by treatment with 5% powdered skim milk (Difco) in TBS (10 mM Tris-HCl, pH 7.4, 150 mM NaCl, 1% bovine serum albumin) containing 0.1% Tween 20 (TBST). Following washings with TBST, membranes were incubated with primary antibodies dissolved in 1% powdered skim milk in TBST for 16 h at 4°C. The following antibodies were used for immunodetection of Mca1-TAP (or its mutant derivatives) and α-tubulin: polyclonal anti-mouse IgG antibody (ICN Biomedicals) and monoclonal anti-α-tubulin antibody (clone B-5-1-2; Sigma-Aldrich), respectively. Following incubation, the membranes were washed and incubated with the appropriate horseradish peroxidase-conjugated secondary antibodies (Amersham Biosciences), developed with enhanced chemiluminescence (ECL) reagents (Amersham Biosciences), and visualized by chemiluminescence using an ImageQuant LAS 4000 instrument (GE Healthcare) equipped with a Fujifilm High Sensitivity F0.85 43 mm camera.

### ChIP assays

*In vivo* cross-linking of proteins was performed by incubating cell cultures with 1% formaldehyde for 20 min at the indicated time after meiotic induction. After formaldehyde-mediated cross-links and neutralization with glycine, cell lysates were prepared by glass bead disruption in lysis buffer containing 100 mM HEPES-KOH pH 7.5, 1% Triton X-100, 0.1% Na-deoxycholate, 1 mM EDTA, 140 mM NaCl, 2X cOmplete ULTRA Tablets (protease inhibitors, Roche), 1 mM phenylmethylsulfonyl fluoride, 50 mM NaF and 0.2 mM Na_3_VO_4_, as described previously [33]. Samples were then sonicated using a Branson 450 sonicator to shear chromatin DNA into fragments of ~500 to 1000 bp. Immunoprecipitation of Mca1-TAP and its mutant derivatives bound to chromatin was performed using immunoglobin G (IgG)-Sepharose beads. In the case of the RNA polymerase II, immunoprecipitation was carried out using protein G Sepharose beads that were coupled to a monoclonal anti-Rpb1 antibody (clone 8WG16, Covance), which specifically recognizes a repeated seven-residue motif (YSPTSTS) located at the C-terminus of Rpb1, termed the C-terminal domain (CTD). In the case of Cuf1-Myc_12_, immunoprecipitation was performed using protein G Sepharose beads that were coupled to a monoclonal anti-myc antibody (9E10, Roche). Handling of beads, including washings and elution, reversed cross-linking, and DNA precipitation were performed as described previously [34, 35]. Quantification of immunoprecipitated DNA was carried out by real-time PCR (qPCR) using different sets of primers that spanned *mfc1*^*+*^, *cuf2*^*+*^, and *ctr4*^*+*^ promoter or coding regions. Primers for ChIP assays were designated by the name of the gene, followed by the position of their 5’ ends relative to that of the translational start codon: mfc1-183 (5’-GTATCGCTAAACTCCGAGGATATAAGTG-3’), mfc1-32 (5’- GAAGATTGAGAATGATGAGAATATATATATCATTTGC-3’), mfc1+875 (5’-TCGTTGGCACTGTTTATGGA-3’), mfc1+960 (5’-TCCCGAAGTGAAGTGGTATTG-3’), cuf2-1139 (5’-TCAGCATCTAGCAGCAAATATACA-3’), cuf2-999 (5’-CGAAGTGATTTCATCAAATTAAGGAACG-3’), ctr4-737 (5’-GAAATGTCACTGTTTGTGATGCTG-3’), and ctr4-584 (5’-CAGCTCTTTTACACGGCAATAATAC-3’). Mca1-TAP, Cuf1-Myc_12_ or RNA polymerase II density at *mfc1*^*+*^, *cuf2*^*+*^, and *ctr4*^*+*^ genes was calculated as the enrichment of specific genomic *mfc1*^*+*^, *cuf2*^*+*^, and *ctr4*^*+*^ DNA regions relative to a 18S ribosomal DNA coding region in which no functional TCGGCG or CuSE element was present. The primers (denoted 18S-a and 18S-b) that were used to amplify a 18S ribosomal DNA region were described previously [7]. Each qPCR was run in triplicate using Perfecta SYBR Green Fast mix (Quanta) on a LightCycler 96 Real-Time PCR instrument (Roche). All ChIP experiments were repeated at least three times using independent chromatin preparations.

## Results

### Mca1 binds to the *mfc1*^*+*^ promoter *in vivo* in a copper-independent manner

**Fig 1:**
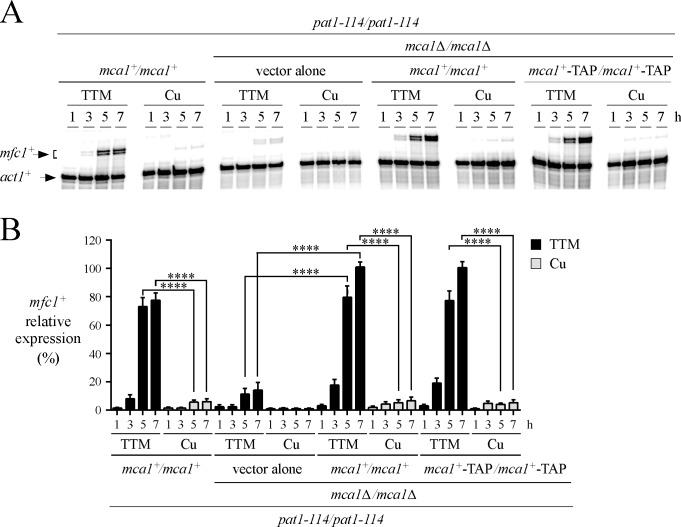
Mca1 is required for maximal copper-starvation-induced expression of *mfc1*^+^. *A*, *pat1-114*/*pat1-114 mca1∆*/*mca1∆* cells containing an empty integrative vector (vector alone) and re-integrated untagged *mca1*^*+*^/*mca1*^*+*^ and *mca1*^*+*^*-TAP*/*mca1*^*+*^*-TAP* alleles were synchronously induced to undergo meiosis under copper-starved (TTM, 150 μM) and copper-replete (Cu, 25 μM) conditions. Total RNA was isolated from culture aliquots taken at the indicated time points after meiotic induction. Following RNA isolation, *mfc1*^*+*^ and *act1*^*+*^ steady-state mRNA levels were analyzed by RNase protection assays. *pat1-114*/*pat1-114 mca1*^*+*^/*mca1*^*+*^ is the isogenic parental strain that was used as a control. *B*, Graphic representation of the quantification of the results of three independent RNase protection assays, including assays shown in panel *A*. Values are represented as averages ± S.D. The asterisks correspond to p<0.0001 (****) (paired Student’s *t*-test).

We have previously reported that the transcription factor Mca1 was required for maximal induction of *mfc1*^*+*^ mRNA levels in response to low concentrations of copper (Fig 1). Despite its transcriptional regulatory role, *mfc1*^*+*^ promoter occupancy *in vivo* by Mca1 remained uncharacterized. To understand the extent by which Mca1 occupies the promoter of *mfc1*^*+*^ *in vivo* under copper starvation compared to its occupancy in the presence of copper, we used a *pat1-114/pat1-114 mca1∆/mca1∆* mutant strain that had been transformed with a control integrative plasmid or a plasmid harboring an untagged or a TAP-tagged version of Mca1 under the control of its own promoter. *pat1-114/pat1-114 mca1*^*+*^*/mca1*^*+*^ (control) and *pat1-114/pat1-114 mca1∆/mca1∆* diploid cells expressing the indicated integrative plasmid (empty, *mca1*^*+*^ or *mca1*^*+*^*-TAP*) were induced to undergo synchronous meiosis in the presence of the copper chelator TTM (150 μM) or CuSO_4_ (25 μM). Subsequently, total RNA was isolated from culture aliquots taken at different time points. In the case of *mca1∆/mca1∆* cells expressing Mca1 or Mca1-TAP, *mfc1*^*+*^ transcripts were initially detected after 3 h of meiotic induction under low copper. These observations contrasted with the absence of significant induction of transcription of *mfc1*^*+*^ mRNA in cells harboring an empty plasmid at the 3-h time point under similar conditions (Fig 1). Results showed that, under copper-starved conditions, *mfc1*^*+*^ mRNA levels reached high levels after 7 h of meiotic induction in *mca1∆/mca1∆* cells expressing an untagged or a TAP-tagged version of Mca1 (7.2-fold) in comparison with cells transformed with the vector alone (Fig 1). The expression profile of *mfc1*^*+*^ mRNA in *mca1∆/mca1∆* cells expressing Mca1 or Mca1-TAP was similar to that observed in the case of *mca1*^*+*^*/mca1*^*+*^ (control) cells, except that the magnitude of induction of *mfc1*^*+*^ mRNA was modestly higher (9%, 3%, and 23% at 3-, 5- and 7-h time points, respectively) (Fig 1). Under copper-replete conditions, transcript levels of *mfc1*^*+*^ were expressed to near background levels in *mca1*^*+*^*/mca1*^*+*^ or *mca1∆/mca1∆* cells whether the cells contained an empty vector or an integrative vector expressing Mca1 or Mca1-TAP (Fig 1).

**Fig 2:**
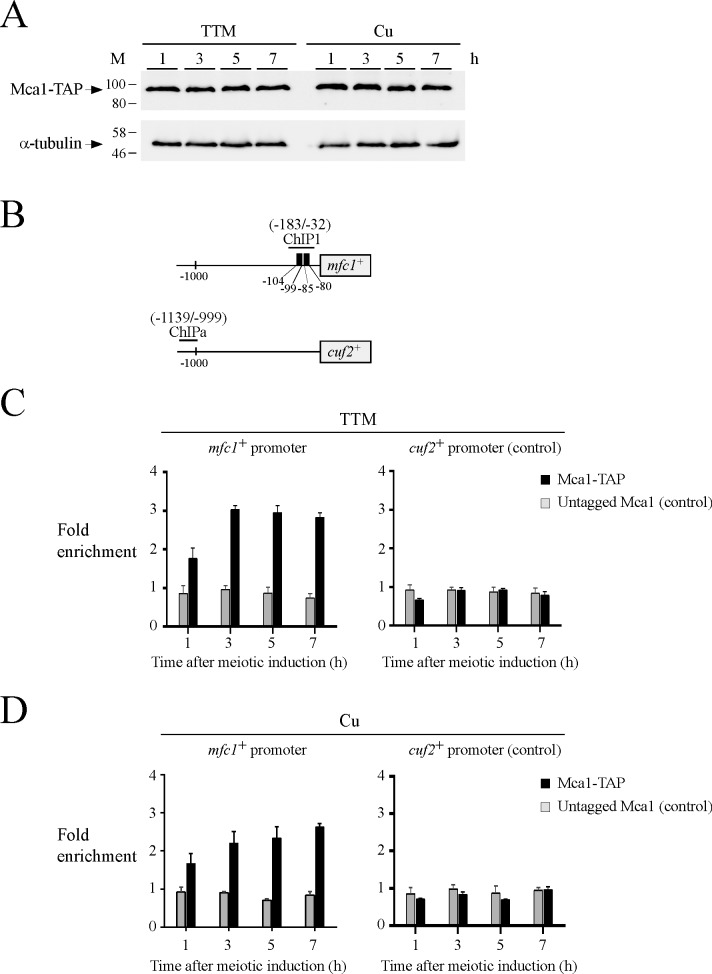
Mca1 constitutively binds the *mfc1*^+^ promoter in vivo. *A*, *pat1-114*/*pat1-114 mca1∆*/*mca1∆* cells expressing Mca1-TAP were synchronously induced into meiosis in the presence of TTM (150 μM) or copper (Cu, 25 μM). The upper panel shows Western blots of Mca1-TAP and α-tubulin protein levels at different time points after meiotic induction. Positions of the molecular weight standards (M) are indicated to the left. *B*, Schematic representation of *mfc1*^*+*^ and *cuf2*^*+*^ promoter regions. Nucleotide numbers refer to the position relative to the A of the initiator codon of each gene (*mfc1*^*+*^ or *cuf2*^*+*^). Numbers in parentheses indicate promoter regions that were used for qPCR analysis in ChIP assays. Black boxes depict wild-type TCGGCG sequences known to serve as a binding site for Mca1. *C–D*, *pat1-114*/*pat1-114 mca1∆*/*mca1∆* cells expressing Mca1-TAP or untagged Mca1 were synchronously induced to undergo meiosis. Following induction of meiosis, chromatin was immunoprecipitated using Sepharose-bound anti-mouse IgG antibodies at the indicated time points. Specific regions of *mfc1*^*+*^ and *cuf2*^*+*^ promoters were analyzed by qPCR to determine Mca1-TAP occupancy in the presence of TTM or copper. Binding of Mca1-TAP to promoters was calculated as the enrichment of specific *mfc1*^*+*^ and *cuf2*^*+*^ promoter regions relative to a 18S ribosomal DNA coding region. The *cuf2*^*+*^ promoter served as a negative control. ChIP data were calculated as values of the largest amount of chromatin measured (fold enrichment). Results are shown as the averages ± S.D. of a minimum of three independent experiments.

Results showed that the in-frame TAP insertion did not interfere with Mca1 function. For example, when Mca1-TAP was expressed in *mca1∆/mca1∆* cells, it conferred copper starvation-dependent regulation of *mfc1*^*+*^ expression in a fashion similar to that of the untagged Mca1 protein. This observation was taken as evidence that Mca1-TAP retained wild-type function. Using *mca1∆/mca1∆* cells expressing Mca1-TAP, we first determined the steady-state protein levels of Mca1-TAP over a time period of 1 to 7 h after meiotic induction. Results showed that Mca1-TAP levels were constitutively present irrespective of the cellular copper status (Fig 2). We took advantage of the constitutive expression of Mca1-TAP to test whether Mca1-TAP could be detected at the *mfc1*^*+*^ and *cuf2*^*+*^ promoters *in vivo* using a ChIP approach. *pat1-114/pat1-114 mca1∆/mca1∆* diploid cells expressing either an untagged or a TAP-tagged Mca1 were synchronized to initiate and proceed through meiosis under copper-deficient or copper-replete conditions. After 1, 3, 5, and 7 h of meiotic induction, results showed that Mca1-TAP constitutively occupied the *mfc1*^*+*^ promoter (Fig 2). Although results showed that Mca1-TAP chromatin occupancy of *mfc1*^*+*^ promoter was slightly higher in copper-starved cells compared to copper-replete cells, Mca1-TAP clearly occupied the *mfc1*^*+*^ promoter under both low and high copper conditions (Fig 2). At the 1-h time point, Mca1-TAP level occupancy was lower in comparison to level observed after 3, 5, and 7 h of meiotic induction. This reduced association of Mca1-TAP with the *mfc1*^*+*^ promoter may contribute to the markedly reduced transcription of *mfc1*^*+*^ in cells at this meiotic time point under low and high copper concentrations (Fig 1). Promoter occupancy by Mca1-TAP was detected using primers amplifying a DNA region located between positions -183 and -32 relative to the initiator codon of *mfc1*^*+*^. This amplified promoter region is known to contain two functional TCGGCG elements that are bound by Mca1 *in vitro* [16]. Results from ChIP analysis showed that Mca1-TAP did not associate with the *cuf2*^*+*^ promoter (used as a negative control). Only very weak levels of *cuf2*^*+*^ promoter fragments were immunoprecipitated (Fig 2). These low levels of immunoprecipitated chromatin were similar to the background signals observed when ChIP assays were performed in *mca1∆*/*mca1∆* cells expressing untagged *mca1*^*+*^, which had been re-integrated (Fig 2). Taken together, these results showed that Mca1 is recruited to the promoter of *mfc1*^*+*^ in a constitutive manner, with a slight increased ability to bind *mfc1*^*+*^ promoter under conditions of low levels of copper.

### The RNA polymerase II is recruited to *mfc1*^*+*^ locus *in vivo* in response to copper starvation

**Fig 3:**
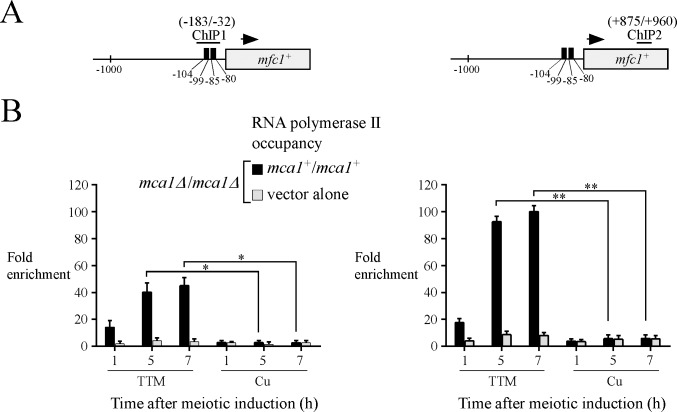
Mca1 is required for copper starvation-dependent RNA polymerase II chromatin occupancy at the *mfc1*^+^ locus after 5 and 7 h of meiotic induction. *A*, Schematic representation of the *mfc1*^*+*^ gene in which ChIP1 and ChIP2 regions indicate locations of the primers that were used for qPCR analysis. Arrows indicate the direction of transcription. Nucleotide numbers refer to the position relative to the A of the initiator codon of *mfc1*^*+*^. *B*, Cultures of *pat1-114*/*pat1-114 mca1∆*/*mca1∆* cells expressing untagged Mca1 or harboring an empty integrative vector were induced to initiate and proceed through meiosis. Cells underwent meiosis in the presence of TTM (150 μM) or copper (Cu, 25 μM). At the indicated times after meiotic induction, chromatin was immunoprecipitated using antibodies against RNA Pol II CTD. ChIP results (RNA Pol II occupancy) are presented as enrichments of specific regions (ChIP1 and ChIP2) relative to a 18S ribosomal DNA coding region. Data were calculated as values of the largest amount of chromatin measured (*fold enrichment*). Results are shown as the averages ± S.D. of a minimum of three independent experiments. The asterisks correspond to p<0.05 (*) and p<0.001 (**) (paired Student’s *t*-test).

The RNA polymerase II (Pol II) is a fundamental multi-subunit enzyme that transcribes numerous genes into mRNA in eukaryotic cells [36]. General, signal- and stress-responsive transcription factors control either positively or negatively gene expression through multiple mechanisms that affect Pol II occupancy at specific coding regions of target genes [37]. Taking into account the result that Mca1 constitutively occupied the *mfc1*^*+*^ promoter, we used a Pol II ChIP assay to determine whether the presence of Mca1 was required for Pol II chromatin occupancy and whether Pol II was primarily recruited in response to low copper availability. Analysis of Pol II chromatin occupancy was performed using an anti-Rpb1 antibody that recognizes the heptapeptide sequence YSPTSPS, which is found in multiple copies at the C-terminal domain (CTD) of Pol II [38]. *pat1-114/pat1-114 mca1∆/mca1Δ* cells harboring an empty integrative plasmid or expressing untagged *mca1*^*+*^ were synchronously induced into meiosis. Immediately before their passage to meiosis, cell cultures were treated with TTM (150 μM) or CuSO_4_ (25 μM). Aliquots of cell cultures were taken 1, 5, and 7 h after meiotic induction and cells were fixed by formaldehyde treatment for ChIP assays. Primers that hybridized immediately upstream (positions -183 to -32) and at the 3’ end of *mfc1*^*+*^ (positions +875 to +960) coding region were used for qPCR analysis (Fig 3A). Results of ChIP analysis showed that in cells expressing Mca1, association of Pol II with the *mfc1*^*+*^ locus was highly enriched in response to copper starvation conditions and was negligible or absent under copper-replete conditions (Fig 3B). In copper-starved cells expressing Mca1, Pol II exhibited 14.0-, 40.1-, and 45.0-fold increases in its binding to *mfc1*^*+*^ (positions -183 to -32) relative to a control 18S ribosomal DNA coding region after 1, 5, and 7 h of meiotic induction (Fig 3B). Under the same conditions, levels of Pol II enrichment were higher (17.6-, 92.4-, and 100.0-fold increases) in its binding at the 3’ end of *mfc1*^*+*^ (positions +875 to +960) coding region relative to a 18S ribosomal DNA region reference (Fig 3B). Taken together, these results revealed that association of Pol II with an *mfc1*^*+*^ transcribed region *in vivo* requires the presence of the transcription factor Mca1 in response to low concentrations of copper.

### The *mfc1*^*+*^ promoter is constitutively bound by Mca1-TAP during mitosis but there is absence of expression of *mfc1*^*+*^ regardless of changes in copper concentrations

**Fig 4:**
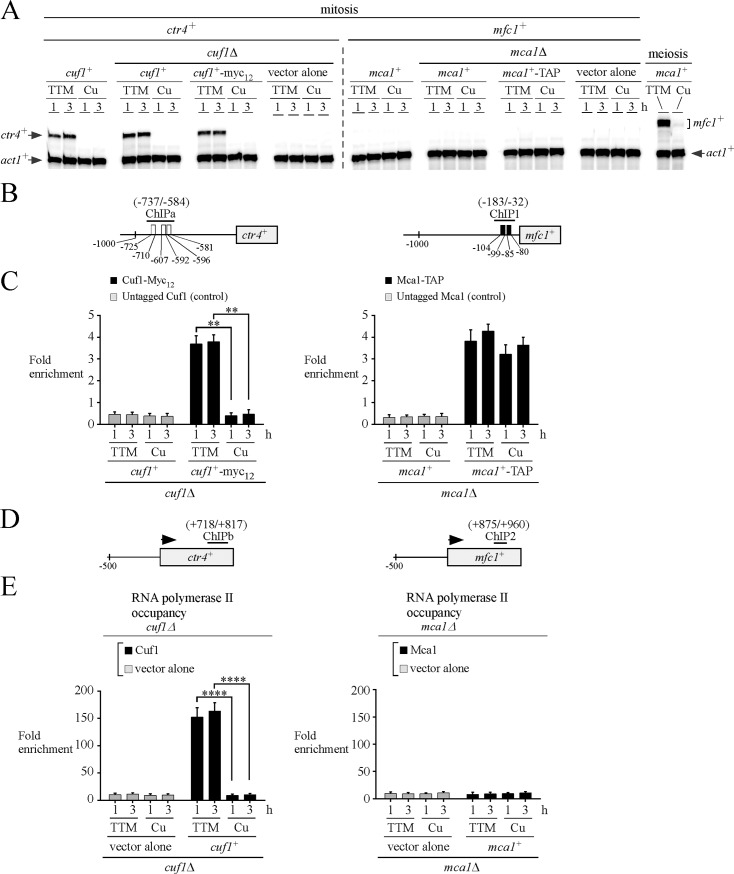
Mca1 interacts with the *mfc1*^+^ promoter when expressed in mitotically growing cells but fails to activate *mfc1*^+^ transcription in response to copper deficiency. *A*, The indicated strains were proliferated in mitosis in the presence of TTM (150 μM) or copper (Cu, 25 μM) for 1 and 3 h. In the cases of *cuf1∆* cells expressing an integrated untagged or a Myc-tagged *cuf1*^*+*^ allele and *mca1∆* cells expressing an integrated untagged or a TAP-tagged *mca1*^*+*^ allele, aliquots of these cultures were fixed by formaldehyde treatment for ChIP assays and at the same time collected to isolate RNA for assessment of transcript levels. Shown are representative RNase protection assays of *ctr4*^*+*^, *mfc1*^*+*^, and *act1*^*+*^ mRNA steady-state levels at the indicated time points. *B*, Schematic representation of *ctr4*^*+*^ and *mfc1*^*+*^ promoter regions. Nucleotide numbers refer to the position relative to the A of the initiator codon of each gene. Numbers in parentheses indicate promoter regions that were used for qPCR analysis in ChIP assays. White and black boxes depict wild-type CuSE and TCGGCG sequences known to serve as binding sites for Cuf1 and Mca1, respectively. *C*, Specific regions of *ctr4*^*+*^ (left) and *mfc1*^*+*^ (right) promoters were analyzed by qPCR to determine chromatin occupancy by Cuf1-Myc_12_ and Mca1-TAP, respectively. Association of Cuf1-Myc_12_ and Mca1-TAP to promoters was calculated as the enrichment of specific *ctr4*^*+*^ and *mfc1*^*+*^ promoter regions relative to a 18S ribosomal DNA coding region. ChIP data were calculated as values of the largest amount of chromatin measured (*fold enrichment*). Results are shown as the averages ± S.D. of a minimum of three independent experiments. *D*, Schematic representation of *ctr4*^*+*^ and *mfc1*^*+*^ coding regions. Numbers in parentheses indicate coding regions that were used for qPCR analysis in ChIP assays. *E*, ChIP analysis was performed to determine RNA Pol II chromatin occupancy at *ctr4*^*+*^ and *mfc1*^*+*^ genes. Chromatin was immunoprecipitated using RNA Pol II CTD-specific antibodies. Data were calculated as values of the largest amount of chromatin measured (*fold enrichment*). Results are shown as the averages ± S.D. of a minimum of three independent experiments. The asterisks correspond to p<0.001 (**) and p<0.0001 (****) (paired Student’s *t*-test).

Previous studies have shown that the copper transporter *ctr4*^*+*^ mRNA levels are induced in a Cuf1-dependent manner in cells proliferating in mitosis under conditions of copper starvation [30, 39, 40]. After treatment with copper, Cuf1 is inactivated [39, 41, 42] and then *ctr4*^*+*^ mRNA is not detected because its expression is dependent of Cuf1 [40]. Here, we used *ctr4*^*+*^ as a control gene known to be induced under copper-limiting conditions (Fig 4A). Furthermore, we performed ChIP assays using Cuf1-Myc_12_ as a control transcription factor known to interact with the *ctr4*^*+*^ promoter *in vivo* under low copper conditions (Fig 4B and 4C) [43]. In the case of the *mfc1*^*+*^ transcription profile, there was no detection of the *mfc1*^*+*^ transcript in mitotically growing cells in the presence or absence of TTM or CuSO_4_ (Fig 4A) [6]. However, the ability of Mca1 to associate with *mfc1*^*+*^ promoter *in vivo* in cells proliferating in mitosis remains unknown. To examine whether there was absence or presence of Mca1 in the promoter region of *mfc1*^*+*^ in mitotic cells, ChIP assays were performed in vegetatively growing *mca1∆* cells expressing a functional *mca1*^*+*^ or *mac1*^*+*^*-TAP* allele under copper-starved or copper-replete conditions. ChIP analysis showed that in the case of cells treated with TTM for 1 and 3 h, Mca1-TAP immunoprecipitated 3.8- and 4.3-fold more chromatin corresponding to *mfc1*^*+*^ promoter compared to an 18S ribosomal region reference (Fig 4B and 4C). In the case of cells exposed to copper for the same times of treatments, chromatin enrichment levels by Mca1-TAP were 3.2- and 3.6-fold, respectively (Fig 4C). These results were consistent with the interpretation that in *mca1∆* cells expressing Mca1-TAP, the *mfc1*^*+*^ promoter was occupied by Mca1-TAP in the presence of TTM in a manner similar to that observed for *mca1∆* cells expressing Mca1-TAP in the presence of copper (Fig 4C; Figure A in S1 File). Results showed that untagged Mca1 (expressed in *mca1∆* cells) immunoprecipitated only background levels of *mfc1*^*+*^ promoter region (Fig 4C). As control experiments, results from ChIP analysis showed that in the case of *cuf1∆* cells expressing Cuf1-Myc_12_ that were treated with TTM for 1 and 3 h, Cuf1-Myc_12_ occupied the *ctr4*^*+*^ promoter with 3.7- and 3.8-fold enrichments, respectively, relative to a control 18S ribosomal DNA region reference (Fig 4C). In these cells, steady-state levels of *ctr4*^*+*^ mRNA consistently increased 11- and 9-fold above the levels observed in copper-replete cells (Fig 4A). In contrast, when cells expressing Cuf1-Myc_12_ were incubated in the presence of copper for 1 and 3 h, only very weak levels of *ctr4*^*+*^ promoter fragments were immunoprecipitated (Fig 4C). These low levels of immunoprecipitated chromatin were similar to the background signals observed when ChIP assays were performed in a *cuf1∆* strain expressing an untagged *cuf1*^*+*^ allele, which had been re-integrated (Fig 4C). The association of Cuf1-Myc_12_ with the *ctr4*^*+*^ promoter was detected using primers amplifying a DNA region located between positions -737 and -584 relative to the initiator codon of *ctr4*^*+*^ (Fig 4B). This amplified promoter region is known to contain functional CuSE elements that are specifically recognized by Cuf1 [30].

Despite a constitutive *mfc1*^*+*^ promoter occupancy by Mca1-TAP, mitotic cells grown under low and high concentrations of copper revealed that the target gene *mfc1*^*+*^ was silent (Fig 4A). To gain insight into this observation, we analyzed Pol II chromatin occupancy using a pair of primers located between positions +875 and +960 relative to the initiator codon of *mfc1*^*+*^. Results showed no enrichment of immunoprecipitated chromatin corresponding to the *mfc1*^*+*^ coding region relative to an 18S ribosomal region reference. The absence of association of Pol II with the *mfc1*^*+*^ locus was observed under low and high concentrations of copper (Fig 4D and 4E). In the case of *ctr4*^*+*^, ChIP experiments showed that Pol II occupied the *ctr4*^*+*^ coding region upstream of the initiator codon of *ctr4*^*+*^ (positions +718 and +817) under copper-limiting conditions. In contrast, in cells undergoing a transition from low to high copper, Pol II was absent from the *ctr4*^*+*^ locus (Fig 4D and 4E). Taken together, these results suggested that cells proliferating in mitosis lacked a factor (perhaps meiosis-specific) or possessed a mechanism that prevented Pol II-dependent transcriptional activation of *mfc1*^*+*^ despite the fact that Mca1 constitutively occupied the *mfc1*^*+*^ promoter *in vivo*.

### *In vivo* mapping of a middle region of Mca1 that is required for induction of *mfc1*^*+*^ expression

**Fig 5:**
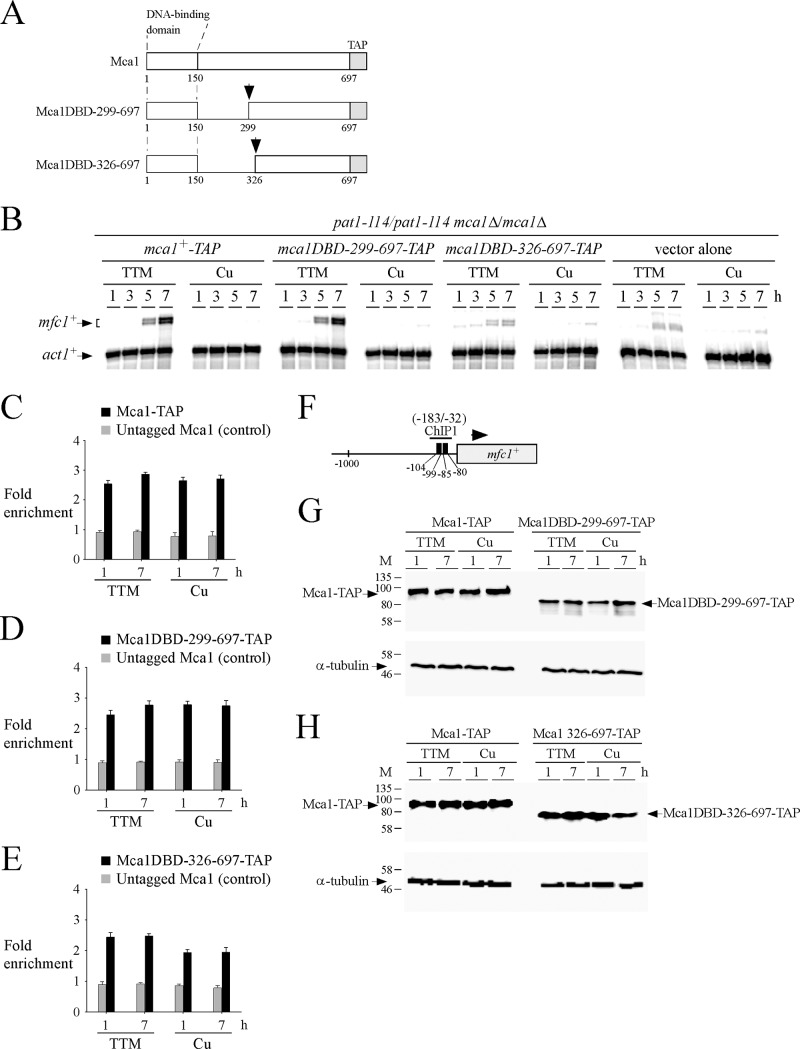
Deletion of the middle region 151–326 of Mca1 inactivates its ability to induce *mfc1*^+^ gene expression in response to low copper. *A*, Schematic diagram of truncated versions of the Mca1-TAP protein. The amino acid sequence of Mca1-TAP is numbered relative to its initiator codon. The black arrow indicates the position of the in-frame deletion within Mca1. *B*, Representative expression profiles of *mfc1*^*+*^ and *act1*^*+*^ transcripts in synchronous meiosis experiments. Mca1-TAP and the indicated mutant proteins were expressed in *pat1-114*/*pat1-114 mca1∆*/*mca1∆* cells and assessed for their ability to activate *mfc1*^*+*^ expression in the presence of TTM (150 μM) or copper (Cu, 25 μM). At the indicated times after meiotic induction, total RNA was isolated and steady-state mRNA levels of *mfc1*^*+*^ and *act1*^*+*^ were analyzed by RNase protection assays. *C–E*, ChIP analysis of the *mfc1*^*+*^ promoter to determine Mca1-TAP, Mca1DBD-299-697-TAP, and Mca1DBD-326-697-TAP chromatin occupancy. TAP-tagged protein density at the *mfc1*^*+*^ promoter was determined as the enrichment of the specific *mfc1*^*+*^ promoter region (positions -183 to -32) relative to a 18S ribosomal DNA coding region. Data were calculated as values of the largest amount of chromatin measured (*fold enrichment*). Results are shown as the averages ± S.D. of a minimum of three independent experiments using distinct chromatin preparations. *F*, Schematic representation of the *mfc1*^*+*^ gene in which ChIP1 region indicates the location of the primers that were used for qPCR analysis. Nucleotide numbers refer to the position relative to the A of the initiator codon of *mfc1*^*+*^. Black boxes depict wild-type TCGGCG sequences known to serve as a binding site for Mca1. *G–H*, Protein extracts were analyzed by Western blots for steady-state protein levels of Mca1-TAP and its mutant derivatives and α-tubulin after 1 and 7 h of meiotic induction.

In cells undergoing synchronous meiosis, Mca1-TAP was constitutively bound to the *mfc1*^*+*^ promoter but recruitment of Pol II and, therefore, *mfc1*^*+*^ expression occurred only under conditions of copper starvation. We thus sought to investigate whether a domain of Mca1 was required for copper starvation-dependent induction of *mfc1*^*+*^. Previous studies had shown that the N-terminal 150-residue segment of Mca1 constituted its DNA-binding domain (DBD) [16]. Because this domain binds DNA constitutively, we kept it intact and generated two internal in-frame Mca1 deletions, starting from Ile^151^ and extending to residues Leu^299^ and Gly^326^, respectively. The rationale for investigating these 2 mutant forms of Mca1 was the fact that the first deleted region (residues 151–298) contains 2 potential metal-binding motifs (Cys^163^-X-Cys-X-His^165^ and Met^234^-X_3_-Met^238^). The resulting C-terminal portions of Mca1 (residues 299–697 and 326–697) were fused in-frame to the DNA binding domain of Mca1 (residues 1–150) (Fig 5A). Because a Mca1-TAP protein triggers activation of *mfc1*^*+*^ gene expression in a manner similar to that of wild-type untagged protein, each mutant derivative was tagged with TAP. The *mca1*^*+*^*-TAP* allele and its mutant derivatives were expressed in *pat1-114/pat1-114 mca1∆/mca1∆* cells that underwent synchronous meiosis under copper-starved or copper-replete conditions. Maximal activation of *mfc1*^*+*^ expression was observed in copper-starved cells expressing *mca1*^*+*^*-TAP* and *mca1DBD-299-697-TAP* alleles after 7 h of meiotic induction (Fig 5B). When the middle region of Mca1 was further deleted to residue 326 to generate the *mca1DBD-326-697-TAP* allele, TTM-dependent expression of *mfc1*^*+*^ was strikingly decreased by 85% to a minimal level of activation that was similar to that observed in the case of cells harboring an empty vector (TTM, 7-h time point) (Fig 5B). In the case of all three alleles (*mca1*^*+*^*-TAP*, *mca1DBD-299-697-TAP*, and *mca1DBD-326-697-TAP*) expressed in *mca1∆/mca1∆* cells, elevated copper concentrations resulted in a strong repression of *mfc1*^*+*^ mRNA expression (Fig 5B).

To further verify that Mca1-TAP and its two mutant derivatives were associated with the *mfc1*^*+*^ promoter *in vivo*, ChIP assays were performed in *pat1-114/pat1-114 mca1∆/mca1∆* cells expressing either an untagged or a TAP-tagged version of Mca1 (wild-type or the indicated mutant version). Enrichment levels of Mca1, Mca1-TAP, Mca1DBD-299-697-TAP, and Mca1DBD-326-697-TAP proteins were assessed at the *mfc1*^*+*^ promoter after 1 and 7 h of meiotic induction and compared to enrichment levels of a 18S ribosomal region reference. Results of ChIP assays showed that chromatin enrichment levels by Mca1-TAP and Mca1DBD-299-697-TAP were similar (with values ranging between 2.5- and 2.8-fold), regardless of the copper status (Fig 5, C–D). In the case of Mca1DBD-326-697-TAP, chromatin enrichment levels were lower by 14% (under TTM conditions) and 30% (under copper conditions) compared to enrichment levels of Mca1-TAP and Mca1DBD-299-697-TAP. However, the *mfc1*^*+*^ promoter was still clearly bound by Mca1DBD-326-697-TAP compared to an untagged Mca1, which immunoprecipitated only background levels of *mfc1*^*+*^ promoter region (Fig 5C and 5E). The association of Mca1-TAP, Mca1DBD-299-697-TAP, and Mca1DBD-326-697-TAP with the *mfc1*^*+*^ promoter was detected using primers amplifying a DNA region located between positions -183 and -32 relative to the initiator codon of *mfc1*^*+*^ (Fig 5F). To assess that Mca1-TAP and its mutant derivatives were produced, total protein extracts were analyzed by immunoblotting at the indicated meiotic time points. Results showed that detectable levels of Mca1-TAP, Mca1DBD-299-697-TAP, and Mca1DBD-326-697-TAP proteins were present in *mca1∆/mca1∆* cells (Fig 5G and 5H).

**Fig 6:**
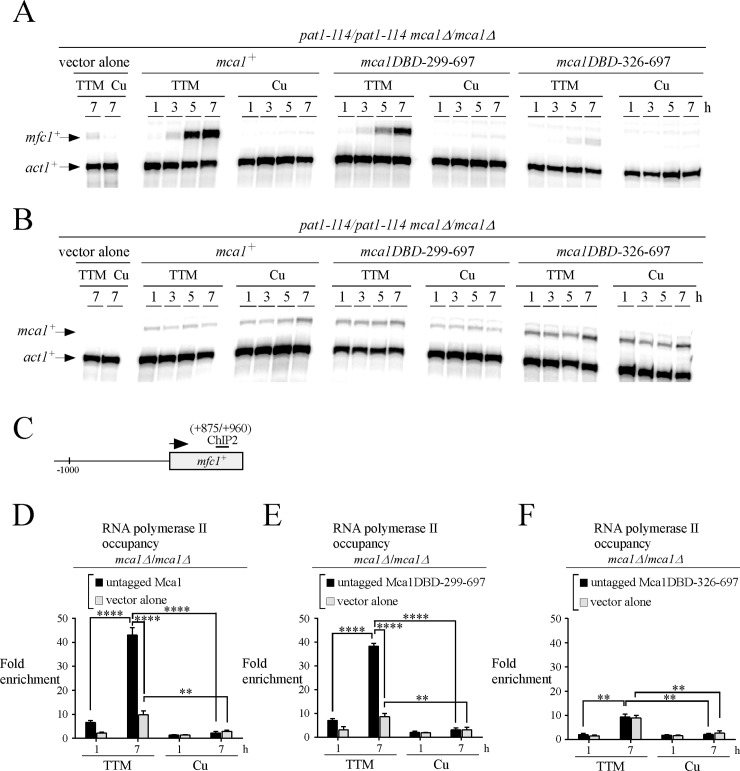
The central deletion 151–326 of Mca1 affects RNA polymerase II chromatin occupancy at the *mfc1*^+^ gene under low copper conditions. *A–B*, *pat1-114*/*pat1-114 mca1∆*/*mca1∆* cells harboring either pBPade6 (vector alone), *mca1*^*+*^*/mca1*^*+*^, *mca1DBD-299-697*/*mca1DBD-299-697* or *mca1DBD-326-697*/*mca1DBD-326-697* alleles were synchronously induced into meiosis. Following induction of meiosis in the presence of TTM (150 μM) or copper (Cu, 25 μM), total RNA was isolated at the indicated time points. After RNA preparation, *mfc1*^*+*^ (*panel A*) and *mca1*^*+*^ (*panel B*) steady-state mRNA levels were analyzed by RNase protection assays using actin (*act1*^*+*^) as an internal control. Results shown are representative of three independent experiments. *C*, Schematic representation of the *mfc1*^*+*^ gene in which the ChIP2 region indicates locations of the primers that were used for qPCR analysis. The arrow indicates the direction of transcription. Nucleotide numbers refer to the position relative to the A of the initiator codon of *mfc1*^*+*^. *D–F*, At the 1 and 7-h meiotic time points, ChIP analysis was performed to determine RNA Pol II chromatin occupancy at the *mfc1*^*+*^ gene. Chromatin was immunoprecipitated using RNA Pol II CTD-specific antibodies. ChIP results are presented as enrichments of *mfc1*^*+*^ (+875/+960) coding region relative to a 18S ribosomal DNA coding region. Data were calculated as values of the largest amount of chromatin measured (*fold enrichment*). Results are shown as the averages ± S.D. of a minimum of three independent experiments. The asterisks correspond to p<0.001 (**) and p<0.0001 (****) (paired Student’s *t*-test).

We next sought to examine whether Mca1, Mca1DBD-299-697, and Mca1DBD-326-697 enhanced Pol II occupancy at the *mfc1*^*+*^ locus. To carry out these experiments, we used untagged Mca1 proteins to avoid any interference of the TAP tag (which can bind to the Fc region of IgG) with the monoclonal anti-RNA Pol II antibody (isotype IgG) that was used for immunoprecipitation of Pol II bound to chromatin. *mca1∆/mca1∆* cells expressing untagged Mca1, Mca1DBD-299-697, and Mca1DBD-326-697 were synchronously induced into meiosis and treated with either TTM (150 μM) or CuSO_4_ (25 μM). Aliquots of cultures were analyzed for their ability to activate *mfc1*^*+*^ transcript levels in response to low concentrations of copper. Results showed that *mca1∆/mca1∆* cells expressing untagged Mca1 and Mca1DBD-299-697 proteins conferred copper starvation-dependent activation of *mfc1*^*+*^ expression in a manner similar to that of TAP-tagged Mca1 and Mca1DBD-299-697 proteins (Figs 5 and 6). *mfc1*^*+*^ mRNA levels were induced within 5 h, exhibiting high levels of expression after 7 h of meiotic induction (Fig 6A). In the case of cells expressing an untagged Mca1DBD-326-697 protein, TTM-dependent transcription of *mfc1*^*+*^ was strongly reduced by 95% to a minimal level of expression that was similar to that observed in the case of cells containing an empty vector (TTM, 7-h time point) (Fig 6A). In the case of all three untagged alleles (*mca1*^*+*^, *mca1DBD-299-697*, and *mca1DBD-326-697*) expressed in *mca1∆/mca1∆* cells, mRNA levels of *mfc1*^*+*^ remained very low under copper-replete conditions (Fig 6A). Given the fact that Mca1 and its mutant derivatives were untagged, we assessed the mRNA levels of the *mca1*^*+*^, *mca1DBD-299-697*, and *mca1DBD-326-697* alleles using aliquots of cultures that served to analyze the expression profile of *mfc1*^*+*^. Results showed that *mca1*^*+*^, *mca1DBD-299-697*, and *mca1DBD-326-697* genes were constitutively expressed as their corresponding transcripts were detected throughout the meiotic program under copper-starved and copper-replete conditions (Fig 6B).

In copper-starved *mca1∆/mca1∆* cells expressing Mca1 and Mca1DBD-299-697, association of Pol II with the *mfc1*^*+*^ locus was enriched 43.0- and 38.2-fold, respectively, relative to a 18S ribosomal region reference (Fig 6C–6E). However, these levels of Pol II enrichment due to the presence of Mca1 and Mca1DBD-299-697 were 33.2- and 29.5-fold when we took into account that Pol II chromatin occupancy was slightly enriched in TTM-treated cells lacking Mca1 (containing an empty vector) after 7 h of meiotic induction (9.8- and 8.7-fold) (Fig 6D and 6E). In the case of copper-starved *mca1∆/mca1∆* cells expressing Mca1DBD-326-697, results showed that Pol II enrichment levels (9.3-fold) were similar to those in *mca1∆/mca1∆* cells containing an empty vector (8.9-fold) (Fig 6F). We therefore concluded that Mca1DBD-326-697 interacted with the *mfc1*^*+*^ promoter *in vivo* (Fig 5E). However, this mutant was unable to foster a significant recruitment of Pol II at the *mfc1*^*+*^ locus, resulting in low levels of *mfc1*^*+*^ mRNA expression even under low concentrations of copper (Fig 6A and 6F). Under copper-replete conditions, results showed that Pol II immunoprecipitated very low or background levels of *mfc1*^*+*^ DNA coding region when *mca1∆/mca1∆* cells expressed *mca1*^*+*^, *mca1DBD-299-697* or *mca1DBD-326-697* alleles (Fig 6D–6F).

### *In vivo* mapping of a C-terminal region of Mca1 that is required for induction of *mfc1*^*+*^ expression

**Fig 7:**
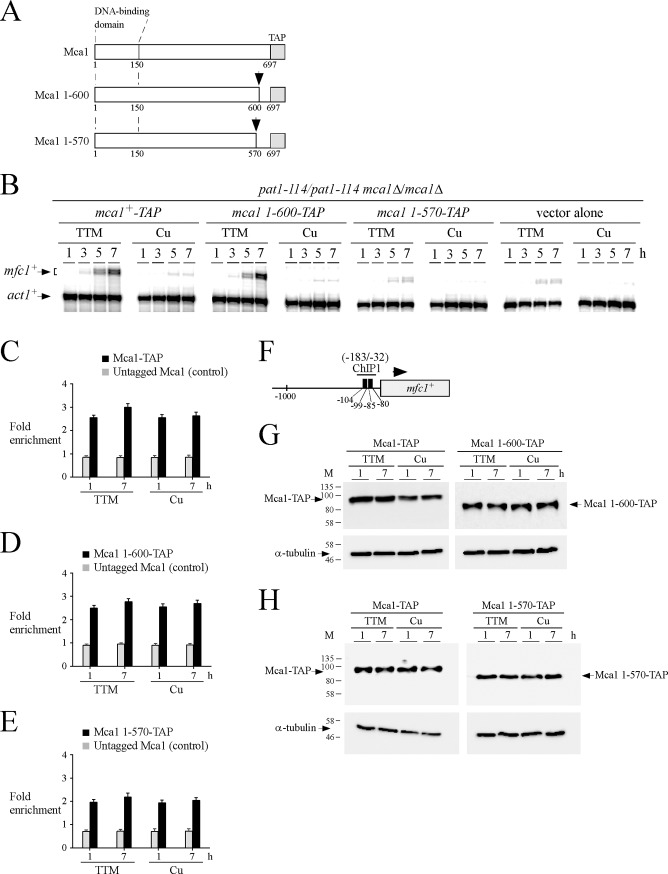
Deletion of the last 127 amino acid residues of Mca1 abolishes its capacity to activate *mfc1*^+^ expression under conditions of copper starvation. *A*, Schematic representation of TAP-tagged Mca1, Mca1 1–600, and Mca1 1–570 proteins. The amino acid sequence of the indicated protein is numbered relative to its initiator codon. The black arrow indicates the position of the in-frame truncation at the C terminus of Mca1. *B*, *pat1-114*/*pat1-114 mca1∆*/*mca1∆* cells expressing TAP-tagged *mca1*^*+*^*/mca1*^*+*^, *mca1 1-600/mca1 1–600*, and *mca1 1-570/mca1 1–570* alleles or an empty vector (vector alone) were synchronously induced to undergo meiosis under copper-depleted (TTM, 150 μM) and copper-replete (Cu, 25 μM) conditions. Total RNA was isolated from culture aliquots taken at the indicated time points. Steady-state levels of *mfc1*^*+*^ and *act1*^*+*^ mRNAs were analyzed by RNase protection assays. Results shown are representative of three independent experiments. *C–E*, ChIP assays were performed from aliquots of cultures used in *panel B* after 1 and 7 h of meiotic induction. Binding of Mca1-TAP, Mca1 1-600-TAP or Mca1 1-570-TAP to the *mfc1*^*+*^ promoter was calculated as the enrichment of the specific *mfc1*^*+*^ proximal regulatory region (positions -183 to -32) relative to a 18S ribosomal DNA coding region. Data were calculated as values of the largest amount of chromatin measured (*fold enrichment*). Results are shown as the averages ± S.D. of a minimum of three independent experiments using separate chromatin preparations. *F*, Schematic representation of the *mfc1*^*+*^ gene in which ChIP1 region indicates the location of the primers that were used for qPCR analysis. Black boxes depict wild-type TCGGCG sequences. *G–H*, Whole-cell extracts were prepared from aliquots of cultures used in *panel B* after 1 and 7 h of meiotic induction. Samples were analyzed by immunoblot assays using anti-IgG and anti-α-tubulin antibodies to detect TAP-tagged proteins and α-tubulin steady-state protein levels, respectively.

To further delineate a region of Mca1 that was required for transcriptional activation of *mfc1*^*+*^ expression under low copper conditions, truncations were created from the C-terminal end of a functional Mca1-TAP protein (Fig 7A). We started with two small deletions due to the fact that the C-terminal end of Zn_2_Cys_6_ cluster transcription factors are known to be required for transcriptional activity of this group of regulators. The wild type *mca1*^*+*^*-TAP* and mutant *mca1 1-600-TAP* and *mca1 1-570-TAP* alleles were returned to *pat1-114/pat1-114 mca1∆/mca1∆* cells. Subsequently, these cells were synchronously induced into meiosis and treated with TTM (150 μM) or CuSO_4_ (25 μM). At the indicated times after meiotic induction, total RNA was isolated from aliquots of cultures and assayed for copper-dependent regulation of the *mfc1*^*+*^ copper-responsive transcript to determine whether the C-terminal end was important for Mca1 function *in vivo*. In the case of *mca1∆/mca1∆* cells expressing Mca1 1-600-TAP under low copper conditions, *mfc1*^*+*^ mRNA levels were first detected at 3- and 5-h time points. RNA levels reached a maximum 7 h after meiotic induction, exhibiting a 15-fold up-regulation compared with *mfc1*^*+*^ transcript levels observed at the same time point (7 h) under copper-replete conditions (Fig 7B). As a control, a similar expression profile of mRNA levels of *mfc1*^*+*^ was observed in *mca1∆/mca1∆* cells expressing Mca1-TAP (Fig 7B). In contrast, *mca1∆/mca1∆* cells expressing the Mca1 1-570-TAP mutant were unable to mediate a robust activation of *mfc1*^*+*^ expression in response to copper starvation. The magnitude of the copper starvation-dependent induction was decreased by 86% and 92% at 5- and 7-h time points, respectively, compared to cells expressing wild-type Mca1-TAP (Fig 7B). In fact, copper-starved *mca1∆/mca1∆* cells expressing Mca1 1-570-TAP exhibited a minimal level of *mfc1*^*+*^ expression that was similar to that observed in the case of cells containing an empty vector (Fig 7B).

To test whether Mca1 1-600-TAP and Mca1 1-570-TAP occupied the *mfc1*^*+*^ promoter region, *pat1-114/pat1-114 mca1∆/mca1∆* cells expressing wild-type Mca1-TAP and these mutant proteins were synchronized to initiate and proceed through the meiotic program. Immediately prior to their entry into meiosis, the cells were either exposed to TTM (150 μM) or CuSO_4_ (25 μM). Aliquots of cultures were retrieved after 1 and 7 h of meiotic induction and ChIP assays were performed to assess the levels of *mfc1*^*+*^ promoter occupancy by Mca1-TAP, Mca1 1-600-TAP, and Mca1 1-570-TAP proteins. Results showed that the *mfc1*^*+*^ promoter was bound by Mca1 1-600-TAP (2.5- and 2.8-fold after 1 and 7 h in the presence of TTM, and 2.3- and 2.7-fold after 1 and 7 h in the presence of CuSO_4_) in a manner similar to that observed for Mca1-TAP (2.5- and 3.0-fold after 1 and 7 h in the presence of TTM, and 2.5- and 2.6-fold after 1 and 7 h in the presence of CuSO_4_) (Fig 7C and 7D). In the case of Mca1 1-570-TAP, its enrichment levels were lower than enrichment levels of Mca1-TAP and Mca1 1-600-TAP (Fig 7E). Notably, Mca1 1-570-TAP constitutively associated with the *mfc1*^*+*^ promoter, exhibiting enrichment levels of 1.9- and 2.2-fold after 1 and 7 h in the presence of TTM, and 1.9- and 2.0-fold after 1 and 7 h in the presence of CuSO_4_ (Fig 7C–7E). The association of Mca1-TAP, Mca1 1-600-TAP, and Mca1 1-570-TAP with the *mfc1*^*+*^ promoter was detected using primers amplifying a DNA region located between positions -183 and -32 relative to the initiator codon of *mfc1*^*+*^ (Fig 7F). To verify that Mca1-TAP, Mca1 1-600-TAP, and Mca1 1-570-TAP proteins were expressed in *pat1-114/pat1-114 mca1∆/mca1∆* cells during the meiotic program, total cell extracts were analyzed by immunoblotting using anti-IgG and anti-α-tubulin antibodies. Results showed that these proteins were present after 1 and 7 h of meiotic induction in the presence of TTM or CuSO_4_ (Fig 7G and 7H).

**Fig 8:**
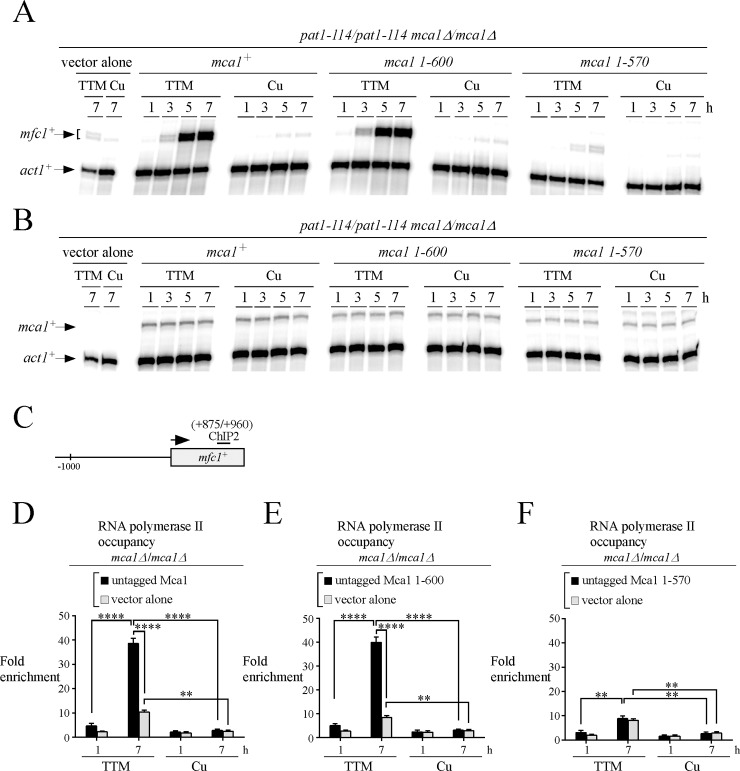
The C-terminal region of Mca1 from residues 570 to 600 is required for RNA polymerase II chromatin occupancy at the *mfc1*^+^ gene. *A–B*, Cultures of *pat1-114/pat1-114 mca1∆/mca1∆* cells expressing either pBPade6 (vector alone), *mca1*^*+*^*/mca1*^*+*^, *mca1 1-600/mca1 1–600* or *mca1 1-570/mca1 1–570* alleles were induced to initiate and proceed through meiosis in the presence of TTM (150 μM) or copper (Cu, 25 μM). At the indicated times after meiotic induction, total RNA was extracted from culture aliquots and steady-state mRNA levels of *mfc1*^*+*^ (*panel A*) and *mca1*^*+*^ (*panel B*) were analyzed by RNase protection assays using actin (*act1*^*+*^) as an internal control. The results shown are representative of three independent experiments. *C*, Schematic representation of the *mfc1*^*+*^ gene in which the ChIP2 region indicates positions of the primers that were used for qPCR analysis. *D–F*, After 1 and 7 h of meiotic induction, aliquots of the cultures described for *panels A* and *B* were taken and chromatin was prepared and immunoprecipitated using antibodies against RNA Pol II CTD. ChIP results are presented as enrichments of *mfc1*^*+*^ (+875/+960) coding region relative to a 18S ribosomal DNA coding region. Data were calculated as values of the largest amount of chromatin measured (*fold enrichment*). Results are shown as the averages ± S.D. of a minimum of three independent experiments. The asterisks correspond to p<0.001 (**) and p<0.0001 (****) (paired Student’s *t*-test).

As mentioned previously untagged version of Mca1, Mca1 1–600, and Mca1 1–570 were created to avoid any interference of the TAP tag with the anti-RNA Pol II antibody (IgG-type) that was used to selectively pull down chromatin bound by Pol II. We examined the individual contribution of each version of the transcription factor (Mca1, Mca1 1–600, and Mca1 1–570) in the regulation of *mfc1*^*+*^ in the presence of TTM (150 μM) or CuSO_4_ (25 μM). Steady-state transcript levels of *mfc1*^*+*^ were analyzed 1, 3, 5, and 7 h after meiotic induction. In the presence of TTM, cells expressing *mca1*^*+*^ and *mca1 1–600* alleles exhibited maximal expression of *mfc1*^*+*^ transcripts that was induced 23- and 25-fold, respectively, compared to their levels of expression in copper-treated cells (7-h time point) (Fig 8A). In the case of cells expressing the *mca1 1–570* allele, the steady-state levels of *mfc1*^*+*^ mRNA were dramatically decreased by 95% under copper-starved (TTM) conditions compared to transcript levels of wild-type Mca1 under the same experimental conditions (7-h time point). Furthermore, the weak levels of *mfc1*^*+*^ transcript in this mutant (Mca1 1–570) were similar to those observed in the case of cells transformed with plasmid alone (Fig 8A).

We assayed the levels of mRNA expression of *mca1*^*+*^, *mca1 1–600*, and *mca1 1–570* alleles by RNase protection assays to obtain further indication that untagged Mca1, Mca1 1–600, and Mca1 1–570 proteins were expressed in *mca1∆/mca1∆* cells. Results showed that wild-type and mutant *mca1* constructs were clearly expressed, with transcripts constitutively detected for each indicated allele regardless of cellular copper status (Fig 8B).

To confirm that Mca1 and Mca1 1–600 affected transcription *per se*, we analyzed Pol II chromatin occupancy at the *mfc1*^*+*^ locus (positions +875 to +960) in *mca1∆/mca1∆* cells expressing *mca1*^*+*^ and *mca1 1–600* alleles. Consistent with the levels of *mfc1*^*+*^ mRNA that were high under low copper conditions at the 7-h time point, levels of Pol II enrichment were higher in cells that had been exposed to TTM after 7 h. Data showed 38.6- (for *mca1*^*+*^) and 40.0-fold (for *mca1 1–600*) enrichment at the *mfc1*^*+*^ locus relative to a 18S ribosomal DNA sequence reference (Fig 8C–8E). In the case of cells expressing the Mca1 1–570 mutant, Pol II enrichment levels were strikingly lower (by 4.5-fold) compared to those observed in *mca1*^*+*^ and *mca1 1–600* cells under the same low copper conditions after 7 h (Fig 8D–8F). In fact, this decrease in the association of Pol II with the *mfc1*^*+*^ locus paralleled a weak expression of *mfc1*^*+*^ (Fig 8A). When meiotic cells were incubated in the presence of copper, there was a lack of Pol II chromatin occupancy at the *mfc1*^*+*^ locus regardless of the expressed allele (*mca1*^*+*^, *mca1 1–600*, and *mca1 1–570*) and meiotic time points (Fig 8D–8F). Taken together, these results indicated that a region of Mca1 encompassing amino acid residues 570 to 600 is required for recruitment of Pol II to the locus of *mfc1*^*+*^. In contrast, the last 97 residues of Mca1 (600–697) are dispensable for Pol II recruitment to chromatin.

### Fusion of amino acid fragments 1–150 and 299–600 of Mca1 are sufficient to trigger proper and timely control of *mfc1*^*+*^ expression

**Fig 9:**
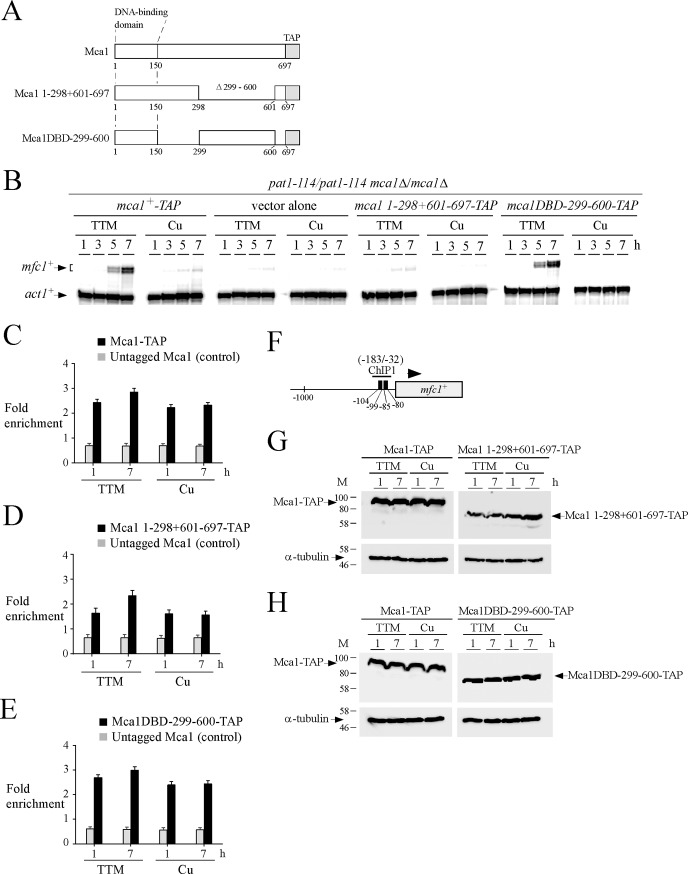
The central region 299–601 of Mca1 is required for copper-dependent regulation of the *mfc1*^+^ gene. *A*, Schematic representation of the Mca1-TAP, Mca1 1–298+601–697, and Mca1DBD–299–600 proteins. The amino acid sequence of the indicated protein is numbered relative to its initiator codon. *B*, *pat1-114*/*pat1-114 mca1∆*/*mca1∆* cells expressing TAP-tagged *mca1*^*+*^*/mca1*^*+*^, *mca1 1–298+601-697/ mca1 1–298+601–697*, and *mca1DBD-299-600/mca1DBD-299-600* alleles or an empty vector (vector alone) were synchronously induced to undergo meiosis under copper-depleted (TTM, 150 μM) and copper-replete (Cu, 25 μM) conditions. Total RNA was isolated from culture aliquots and steady-state levels of *mfc1*^*+*^ and *act1*^*+*^ mRNAs were analyzed at different time points after meiotic induction. Results shown are representative of three independent RNase protection experiments. *C–E*, ChIP assays were performed from aliquots of cultures used in *panel B* after 1 and 7 h of meiotic induction. Binding of Mca1-TAP, Mca1 1–298+601–697-TAP or Mca1DBD–299–600-TAP to the *mfc1*^*+*^ promoter was calculated as the enrichment of the specific *mfc1*^*+*^ proximal regulatory region (positions -183 to -32) relative to a 18S ribosomal DNA coding region. Data were calculated as values of the largest amount of chromatin measured (*fold enrichment*). Results are shown as the averages ± S.D. of a minimum of three independent experiments using separate chromatin preparations. *F*, Schematic representation of the *mfc1*^*+*^ gene in which ChIP1 region indicates positions of the primers that were used for qPCR analysis. Black boxes depict wild-type TCGGCG sequences. *G–H*, Whole-cell extracts were prepared from aliquots of cultures used in *panel B* after 1 and 7 h of meiotic induction. Samples were analyzed by immunoblot assays using anti-IgG and anti-α-tubulin antibodies to detect TAP-tagged proteins and α-tubulin steady-state protein levels, respectively.

Given the results of *mfc1*^*+*^ expression under the control of different Mca1 mutants, we hypothesized that residues 299 to 600 of Mca1 were required for transcriptional activation of *mfc1*^*+*^ expression. Accordingly, disruption of this region would result in poor expression of *mfc1*^*+*^ under copper starvation conditions. To test this hypothesis, we deleted the amino acid region 299 to 600 of Mca1-TAP (Fig 9A). The resulting allele was named *mca1 1–298+601-697-TAP*. As a complementary approach, a chimeric protein that consisted in a fusion between amino acid fragments 1 to 150 (DBD) and 299 to 600-TAP of Mca1 (denoted *mca1DBD-299-600-TAP*) was created to test whether it mediated proper and timely control of *mfc1*^*+*^ gene expression as a function of copper availability. The experiments were set by expressing *mca1*^*+*^*-TAP*, *mca1 1–298+601-697-TAP*, and *mca1DBD-299-600-TAP* alleles into *pat1-114/pat1-114 mca1∆/mca1∆* cells that were synchronously induced into meiosis in the presence of TTM (150 μM) or CuSO_4_ (25 μM). Aliquots of cell cultures were taken 1, 3, 5, and 7 h after meiotic induction, and steady-state levels of *mfc1*^*+*^ mRNA were analyzed by RNase protections assays. Under low copper conditions (TTM), maximal induction of *mfc1*^*+*^ was primarily observed in cells expressing *mca1*^*+*^*-TAP* and *mca1DBD-299-600-TAP* alleles after 7 h of meiotic induction (Fig 9B). In contrast, mRNA levels of *mfc1*^*+*^ remained low (96% reduction) in TTM-treated cells expressing *mca1 1–298+601-697-TAP* or an empty vector (Fig 9B). In the case of all the three indicated alleles expressed in *mca1∆/mca1∆* cells, there was a lack of significant induction of *mfc1*^*+*^ mRNA under copper-replete conditions (Fig 9B).

Despite the fact that there was only copper starvation-dependent induction of *mfc1*^*+*^ expression in cells expressing Mca1-TAP and Mca1DBD-299-600-TAP, we tested whether Mca1-TAP, Mca1 1–298+601-697-TAP, and Mca1DBD-299-600-TAP could bind to the *mfc1*^*+*^ promoter *in vivo* under both low and high concentrations of copper. Results of ChIP analysis showed that in the case of cells treated with TTM after 7 h of meiotic induction, Mca1-TAP, Mca1 1–298+601-697-TAP, and Mca1DBD-299-600-TAP occupied the *mfc1*^*+*^ promoter with 2.8-, 2.3- and 3.0-fold enrichments, respectively, relative to a control 18S ribosomal DNA reference (Fig 9C–9E). Levels of TAP-tagged Mca1, Mca1 1–298+601–697, and Mca1DBD-299-600 enrichments displayed a slight increase at later time point (7 h) compared to earlier time point (1 h) (Fig 9C–9E). We subsequently assessed whether copper affected Mca1-TAP, Mca1 1–298+601-697-TAP, or Mca1DBD-299-600-TAP ability to interact with the *mfc1*^*+*^ promoter *in vivo*. Under copper-replete conditions, results showed that the association of Mca1-TAP, Mca1 1–298+601-697-TAP, and Mca1DBD-299-600-TAP with the *mfc1*^*+*^ promoter was respectively detected with 2.3-, 1.6-, and 2.4-fold enrichment after 7 h of meiotic induction relative to a control 18S ribosomal DNA reference (Fig 9C–9E). These results revealed that only a slight decrease of chromatin occupancy by Mca1-TAP, Mca1 1–298+601-697-TAP, and Mca1DBD-299-600-TAP at the *mfc1*^*+*^ promoter region was observed in copper-treated cells. We also noticed that the association of Mca1 1–298+601-697-TAP with the *mfc1*^*+*^ promoter was weaker than Mca1-TAP or Mca1DBD-299-600-TAP under both copper-limiting and copper-replete conditions. Immunoprecipitated chromatin by Mca1-TAP and its mutant derivatives was detected using primers amplifying a DNA region located between positions -183 and -32 relative to the initiator codon of *mfc1*^*+*^ (Fig 9F). To ensure that Mca1-TAP, Mca1 1–298+601-697-TAP, and Mca1DBD-299-600-TAP proteins were synthesized when expressed in *pat1-114/pat1-114 mca1∆/mca1∆* cells during the meiotic program, whole cell extracts were analyzed by immunoblot assays using anti-IgG and anti-α-tubulin antibodies. Results showed that these proteins were present after 1 and 7 h of meiotic induction in the presence of TTM or CuSO_4_ (Fig 9G and 9H).

**Fig 10:**
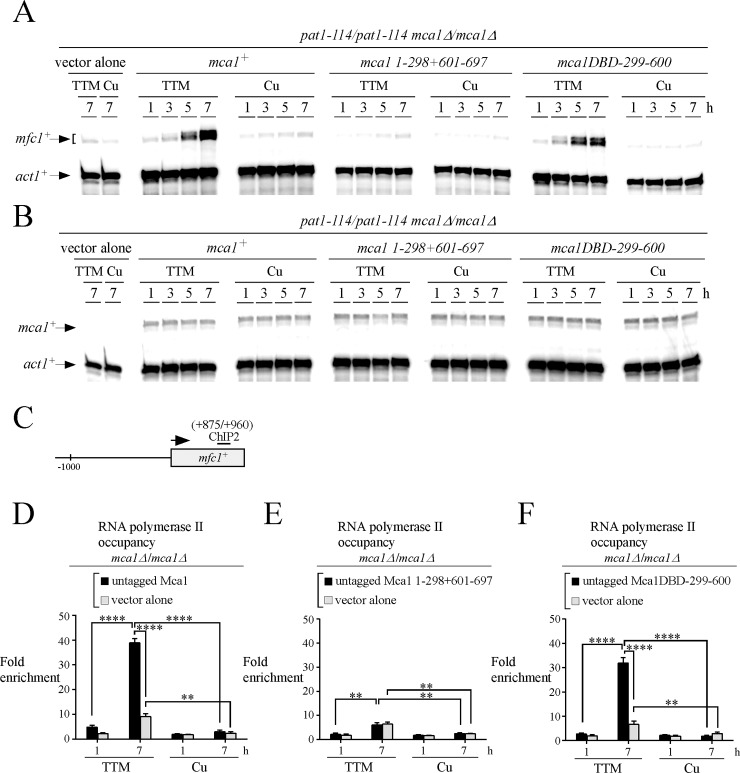
The central segment of Mca1 from amino acids 299–600 is required for RNA polymerase II chromatin occupancy at the *mfc1*^+^ gene. *A–B*, Cultures of *pat1-114/pat1-114 mca1∆/mca1∆* cells expressing an empty plasmid (vector alone), *mca1*^*+*^*/mca1*^*+*^, *mca1 1–298+601-697/ mca1 1–298+601–697*, or *mca1DBD-299-600/mca1DBD-299-600* alleles were synchronously induced to undergo meiosis in the presence of TTM (150 μM) or copper (Cu, 25 μM). At the indicated times after meiotic induction, total RNA was extracted from culture aliquots and steady-state mRNA levels of *mfc1*^*+*^ (*panel A*) and *mca1*^*+*^ (*panel B*) were analyzed by RNase protection assays using actin (*act1*^*+*^) as an internal control. The results shown are representative of three independent experiments. *C*, Schematic representation of the *mfc1*^*+*^ gene in which the ChIP2 region indicates positions of the primers that were used for qPCR analysis. *D–F*, At the 1 and 7-h meiotic time points, aliquots of the cultures described for *panels A* and *B* were taken and chromatin was prepared and immunoprecipitated using antibodies against RNA Pol II CTD. ChIP results are presented as enrichments of *mfc1*^*+*^ (+875/+960) coding region relative to a 18S ribosomal DNA coding region. Data were calculated as values of the largest amount of chromatin measured (*fold enrichment*). Results are shown as the averages ± S.D. of a minimum of three independent experiments using separate chromatin preparations. The asterisks correspond to p<0.001 (**) and p<0.0001 (****) (paired Student’s *t*-test).

Untagged *mca1*^*+*^, *mca1 1–298+601–697*, and *mca1DBD-299-600* alleles were expressed in *pat1-114/pat1-114 mca1∆/mca1∆* cells to allow analysis of Pol II chromatin occupancy at the *mfc1*^*+*^ locus. This strategy was used to avoid any interference from the TAP tag with respect to the enrichment of chromatin-bound Pol II to Protein G Sepharose beads. Before entry into meiosis, cells were exposed to TTM (150 μM) or CuSO_4_ (25 μM). Aliquots of cultures were retrieved at distinct time points after meiotic induction, and steady-state levels of *mfc1*^*+*^, *mca1*^*+*^, and *act1*^*+*^ mRNAs analyzed by RNase protection assays. In the case of cells expressing untagged *mca1*^*+*^ and *mca1DBD-299-600* alleles, *mfc1*^*+*^ transcripts showed an expression profile similar to that observed in the case of cells transformed with *mca1*^*+*^*-TAP* and *mca1DBD-299-600-TAP* (Figs 9B and 10A). Under copper-limiting conditions, *mfc1*^*+*^ mRNA levels were strongly induced after 5 and 7 h of meiotic induction (Fig 10A). Conversely, copper supplementation resulted in strongly reduced *mfc1*^*+*^ mRNA levels in comparison to those observed with cells grown under low copper conditions (Fig 10A). In cells expressing Mca1 1–298+601–697 and an empty vector, transcript levels of *mfc1*^*+*^ remained low, irrespective of cellular copper status (Fig 10A). We next analyzed *mca1*^*+*^ mRNA levels of cells expressing the indicated mutant alleles as a function of copper availability. Results showed that untagged *mca1*^*+*^, *mca1 1–298+601–697*, and *mca1DBD-299-600* alleles were expressed and their steady-state mRNA levels were constitutive and unresponsive to cellular copper status (Fig 10B).

To determine whether the levels of Pol II occupancy at *mfc1*^*+*^ followed transcriptional activation of *mfc1*^*+*^ by Mca1 and Mca1DBD-299-600, we probed Pol II chromatin occupancy using primers covering a coding region of *mfc1*^*+*^ (positions +875 to -960) (Fig 10C). These experiments were performed in *mca1∆/mca1∆* cells expressing untagged *mca1*^*+*^, *mca1 1–298+601–697*, and *mca1DBD-299-600* alleles. Under copper-limiting conditions, results of ChIP analysis showed that Pol II enrichment levels were consistently higher in cells expressing *mca1*^*+*^ (38.9-fold) and *mca1DBD-299-600* (34.8-fold) than those observed in cells expressing *mca1 1–298+601–697* (6.1-fold) after 7 h of meiotic induction (Fig 10D–10F). In contrast, there was no significant Pol II enrichment in cells expressing *mca1*^*+*^, *mca1 1–298+601–697*, and *mca1DBD-299-600* alleles under copper-replete conditions (Fig 10D–10F). Results of chromatin immunoprecipitation experiments of *mca1∆/mca1∆* cells containing an empty vector showed only background levels of Pol II bound to a *mca1*^*+*^ transcribed region (Fig 10D–10F). Taken together, these results revealed that the central segment of Mca1 that comprised amino acid residues 299 to 600 is required for Pol II-mediated maximal induction of *mfc1*^*+*^ expression in copper-limited meiotic cells.

## Discussion

Four proteins have been identified to ensure copper transport in meiotic *S*. *pombe* cells. These proteins are Ctr4, Ctr5, Ctr6, and Mfc1 [6, 15]. The heteromeric cell-surface Ctr4-Ctr5 complex is predicted to play a critical role for the acquisition of the primary pool of copper from the environment shortly after induction of meiosis [15]. In the case of Ctr6, the protein localizes to vacuolar membranes in early meiosis and then undergoes redistribution in a time-dependent manner to reach forespore membranes [15]. Ctr6 serves to mobilize intravacuolar stores of copper and participates in the delivery of copper to the cytosolic copper-dependent enzyme Sod1 [15, 44]. Ctr4/5 and Ctr6 are expressed in mitosis and at precise stages of meiosis, whereas Mfc1 is exclusively expressed during meiosis [6, 15]. Mfc1 is transcriptionally induced throughout meiotic divisions and spore maturation process under low copper conditions. After meiotic divisions and FSM closure, Mfc1 resides at the FSM where it mediates copper accumulation into prespores [6]. As opposed to the copper starvation-dependent expression profiles of *ctr4*^*+*^, *ctr5*^*+*^, and *ctr6*^*+*^ transcripts that are regulated by Cuf1, the induction of *mfc1*^*+*^ gene expression is primarily dependent of Mca1 [16]. When the *mfc1*^*+*^ promoter was analyzed by ChIP assays, results showed that maximal transcriptional induction of *mfc1*^*+*^ was observed in response to copper deficiency and that Mca1 was constitutively bound to the *mfc1*^*+*^ promoter irrespective of changes in copper concentrations. Additional *mfc1*^*+*^ ChIP analysis showed that optimal induction of *mfc1*^*+*^ transcription coincided with maximal chromatin occupancy by RNA Pol II under low copper conditions. In contrast, there was a lack of RNA Pol II recruitment at the *mfc1*^*+*^ locus in response to excess copper.

The fact that Mca1 was constitutively bound to its target DNA sites upstream of *mfc1*^*+*^ was reminiscent of the situation observed for other members of the Zn_2_Cys_6_ binuclear cluster family of transcription factors. In *S*. *cerevisiae*, the zinc cluster transcriptional activator Put3 is constitutively bound to its DNA-recognition sites [45, 46]. In the presence of proline as the sole source of nitrogen, there is a conversion of DNA-tethered Put3 from a transcriptionally inert form to a transcriptionally active form [46, 47]. Accordingly, the presence of proline is predicted to induce a conformational change in the DNA-bound form of Put3, thereby unmasking its activation domain and enabling the recruitment of the RNA Pol II transcriptional machinery [46]. The *S*. *cerevisiae* Gal4 protein is another example of zinc cluster transcription factor that is constitutively bound to *cis*-acting regulatory elements upstream of the *GAL* genes when galactose is present as the sole source of carbon [48]. The DNA-binding activity of Gal4 does not play a major role in controlling transcriptional activation of the *GAL* genes by Gal4. In the absence of galactose, the activity of DNA-bound Gal4 is inhibited by its association with the transcriptional repressor Gal80 [46, 49]. Conversely, in cells undergoing a transition from a glucose to galactose source, the galactose sensor Gal3 becomes active and interacts with the transcriptional repressor Gal80. The net result of Gal3-Gal80 association is that Gal4 becomes active and induces the expression of the *GAL* genes [46, 50]. In the case of chromatin-bound Mca1, its ability to induce transcription of *mfc1*^*+*^ in response to copper deficiency occurred only during meiosis but not in cells undergoing mitotic growth. This observation raises the possibility that a meiosis-specific transcriptional inducer may trigger an active state of DNA-bound Mca1. An alternative possibility is that a meiosis-specific factor may inactivate an inhibitory partner of Mca1. In both cases, this would result in the freeing of Mca1 from an inactive state, resulting in transcriptional activation of *mfc1*^*+*^ to occur during specific stages of the meiotic differentiation program. This mechanism would require the meiosis-specific transcriptional inducer to respond to changing environmental copper concentrations because its function is required to induce a switch in Mca1 transcriptional activity to regulate *mfc1*^*+*^ expression as a consequence of copper starvation. Another potential mechanism could be that copper-dependent alterations in Mca1 activity might arise from the formation of a heterodimer complex between Mca1 and a second related Zn_2_Cys_6_ cluster transcription factor.

We have identified an internal region (residues 299 to 600) of Mca1 that was required for RNA Pol II-dependent timely activation of the meiotic *mfc1*^*+*^ gene in response to low copper concentrations. Interestingly, this internal region of Mca1 could be aligned with a region encompassing two inhibitory domains, called ID2 and ID3, that have been identified in the *S*. *cerevisiae* Gal4 protein [21, 22]. Although the role of Gal4 ID2 and ID3 domains is not well understood, it is thought that they may participate in two functions of Gal4. First, ID2 and ID3 may play a role in restricting transactivation in the absence of activating cues. Second, after relieving DNA-bound Gal4 inhibition, the internal region of Gal4 (containing ID2 and ID3) may serve as a spacer to augment transcription and/or it may be involved in protein-protein interactions with transcriptional coactivators to promote target gene activation. In the case of *S*. *cerevisiae* Gal4, the transcriptionally active form of DNA-bound Gal4 interacts directly with the SAGA co-activator-histone-modifying complex, specifically with the Tra1 subunit [51, 52]. Other subunits of SAGA such as Spt3 and Spt8 associate directly with components of the RNA Pol II Mediator complex, which contains a number of highly conserved subunits that can interact directly with RNA Pol II, leading to the formation of a RNA Pol II holoenzyme [53]. Based on these results in *S*. *cerevisiae*, one can envision that activation of the transcriptional properties of DNA-bound Mca1 may also involve successive series of interactions with components of SAGA, the fission yeast Mediator complex, and the RNA Pol II, leading to the formation of a competent RNA Pol II holoenzyme for regulated expression of *mfc1*^*+*^.

Adjacent to the internal ID2- and ID3-like domains of Mca1, an ID1-like region encompassing amino acid residues 151 to 298 was removed (Mca1DBD-299-697 mutant) to test whether this deletion would affect the function of Mca1. Results showed that the Mca1DBD-299-697 mutant protein was active and yielded appropriate timely meiotic induction of *mfc1*^*+*^ gene expression under low copper conditions. As observed in the case of cells expressing the wild-type protein and mutant cells expressing the *mca1DBD-299-697* allele in the presence of copper, results showed lack of inducing *mfc1*^*+*^ transcription. These results suggested that the ID1-like region of Mca1 was not required for maintaining Mca1 transcriptionally inactive under copper-replete conditions. The bulk of these observations led us to conclude that ID2- and ID3-like domains but not the ID1-like region, could be considered as a middle homology region (MHR) with a role in regulating the transcriptional activity of Mca1.

As of now, it is not known how limiting copper concentrations can trigger a signal for activation of *mfc1*^*+*^ gene expression in a Mca1-dependent manner. In the case of the copper-sensing transcription factor Cuf1, a highly conserved C-terminal motif containing the Cys^328^-X-Cys^330^-X_3_-Cys^334^-X-Cys^336^-X_2_-Cys^339^-X_2_-His^342^ sequence (termed C-rich) constitutes its copper-sensing region [39]. A current model for activation by Cuf1 posits that, the copper-free form of Cuf1 binds to its target DNA sequences (denoted copper-signaling elements) within the promoter of the regulated genes to induce transcription. In contrast, when copper is in excess, Cuf1 is sequestered in the cytoplasm through a process that requires the presence of its C-terminal C-rich sequence, leading to cytosolic accumulation of Cuf1 [41]. Furthermore, excess copper promotes an interaction between the Cuf1 N terminus and its C terminal region encompassing the C-rich sequence. This intramolecular interaction fosters the shutoff of Cuf1 in two ways. First, Cuf1 is inhibited from entering the nucleus. Second, the pre-existing nuclear pool of Cuf1 is exported from the nucleus to the cytoplasm, resulting in down regulation of Ctr4/5/6 gene expression [41, 42]. In the case of Mca1, the protein does not contain a C-rich-like motif, which suggests that Mca1 regulates *mfc1*^*+*^ gene expression through a mechanism different from that of Cuf1. Within the internal region of Mca1 that encompasses amino acid residues 299 to 600, two potential metal-binding motifs (Cys^426^-X_3_-Met-His-X_3_-His^478^ and Met^476^-X-Met^478^) are present. We have examined the potential role of these motifs in copper starvation-dependent regulation of *mfc1*^*+*^ by generating mutant alleles of *mca1*^*+*^ in which all of the Cys, Met, and His residues had been mutated to alanines. Each Mca1 mutant protein containing either the Ala^426^-X_3_-Ala-Ala-X_3_-Ala^478^ or Ala^476^-X-Ala^478^ mutated motif had no effect on the expression of the *mfc1*^*+*^ gene as compared to that of TTM-mediated activation of *mfc1*^*+*^ transcription in cells expressing a wild-type *mca1*^*+*^ allele (Figure B in S1 File).

Due to the fact that Mca1 was constitutively bound to its target promoter, modulation of the DNA-binding activity of Mca1 does not appear to play a major role in controlling the activity of the genetic switch in response to induction of *mfc1*^*+*^ by copper starvation. Mechanisms that modulate transcriptional activities of members of the zinc cluster transcription factor family include interaction with small-molecule metabolites, modification of phosphorylation state, and recruitment of co-regulators and partner nutrient sensors [17, 20]. Because an internal region (residues 299 to 600) of Mca1 contributes to the activation of *mfc1*^*+*^ in response to copper starvation, it is likely that an unidentified meiosis-specific factor or timely posttranslational modification mediates a conformational change in the Mca1 protein required to convert Mca1 from a transcriptionally inert form to a transcriptionally active form, leading to induction of *mfc1*^*+*^ expression in response to copper deficiency.

## Supporting information

S1 File Figure A. Western blots of Mca1-TAP and α-tubulin protein levels when cells proliferated in mitosis in the presence of the copper chelator TTM or copper.
Figure B. Assessment of mRNA levels of mfc1+ in pat1-114/pat1-114 mca1∆/mca1∆ cells expressing mutated mca1Ala426-X3-Ala-Ala-X3-Ala478-TAP and mca1Ala476-X-Ala478 alleles under copper-limiting and copper-replete conditions.
(DOC)

## Abbreviations

bp: base pair(s)
ChIP: chromatin immunoprecipitation
Ctr: copper transporter
Cuf1: copper factor 1
DBD: DNA-binding domain
EMM: Edinburgh minimal medium
FSM: forespore membrane
GFP: green fluorescent protein
Mfc1: major facilitator copper transporter 1
MHR: middle homology region
ORF: open reading frame
PCR: polymerase chain reaction
Pol II: RNA polymerase II
qPCR: real-time polymerase chain reaction
TTM: ammonium tetrathiomolybdate
YES: yeast extract plus supplements
WT: wild-type
