# Supplementary material for: A role for the transcription factor Mca1 in activating the meiosis-specific copper transporter Mfc1
Source: PLoS One. 2018 Aug 7;13(8):e0201861. doi: 10.1371/journal.pone.0201861 (PMC6080790; doi:10.1371/journal.pone.0201861)
Supplement: S1 File — Figure A. Western blots of Mca1-TAP and α-tubulin protein levels when cells proliferated in mitosis in the presence of the copper chelator TTM or copper. Figure B. Assessment of mRNA levels of mfc1+ in pat1-114/pat1-114 mca1∆/mca1∆ cells expressing mutated mca1Ala426-X3-Ala-Ala-X3-Ala478-TAP and mca1Ala476-X-Ala478 alleles under copper-limiting and copper-replete conditions. (DOC) [file pone.0201861.s001.doc]

**SUpporting information**

A role for the transcription factor Mca1 in activating the meiosis-specific copper transporter Mfc1.

Jude Beaudoin, Raphael Ioannoni, Vincent Normant, and Simon Labbé^*^.

**
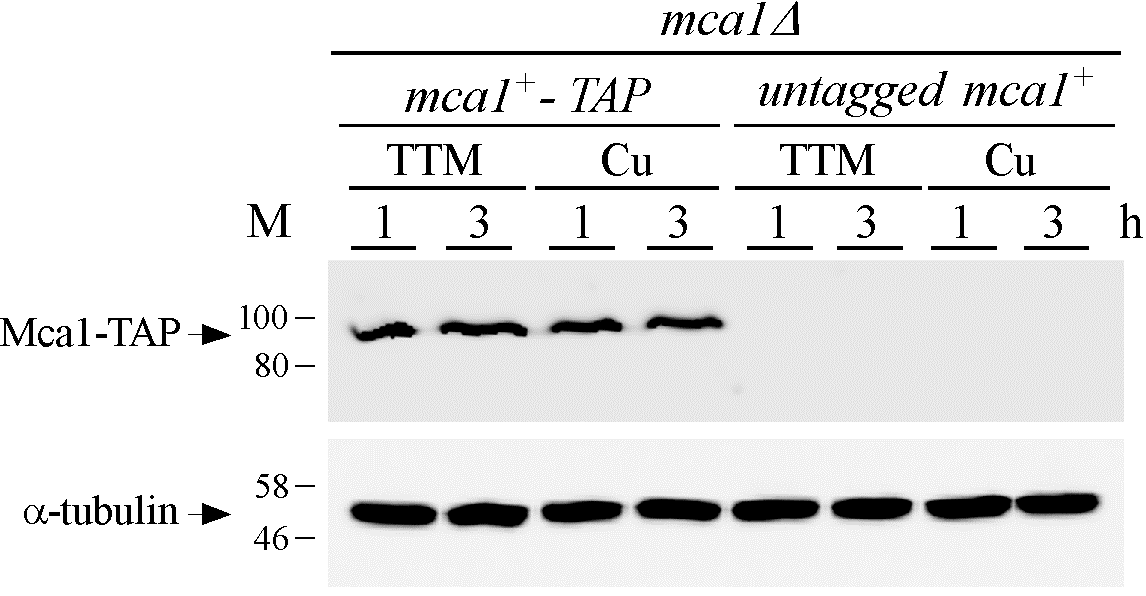
**

**Figure A.** *Western blots of Mca1-TAP and α-tubulin protein levels when cells proliferated in mitosis in the presence of the copper chelator TTM or copper*.

Cell lysates from aliquots of *mca1Δ* cultures expressing a *TAP-tagged mca1^+^* or an untagged *mca1^+^* allele were analyzed by immunoblotting using either anti-IgG or anti-α-tubulin (as an internal control) antibody. Mitotic cells were treated with TTM (150 μM) or CuSO_4_ (25 μM) for 1 or 3 h. Positions of the molecular weight protein standards (M) (in kDa) are indicated on the left-hand side.

**
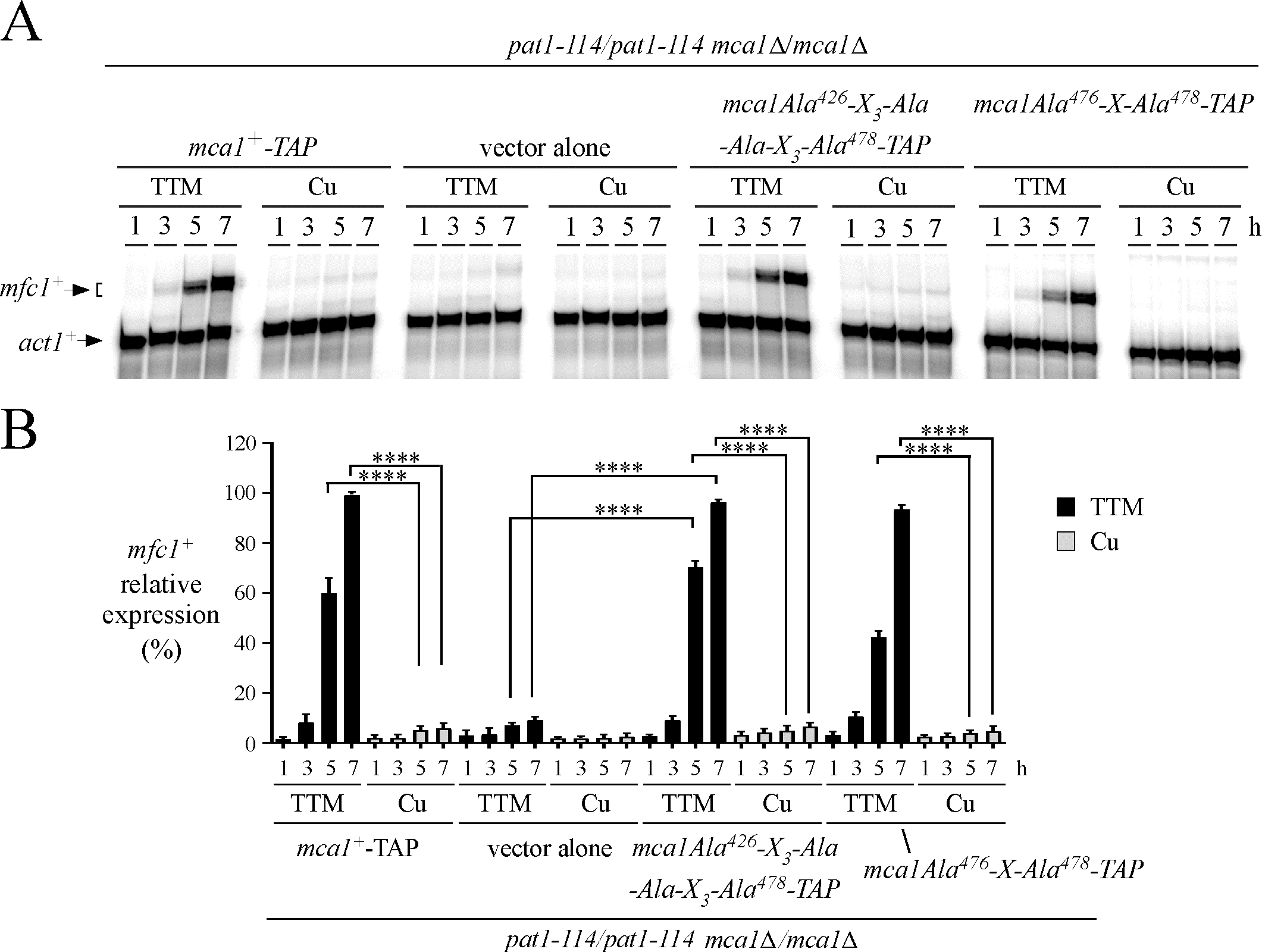
**

**Figure B.** Assessment of mRNA levels of *mfc1^+^* in *pat1-114/pat1-114 mca1Δ/mca1Δ* cells expressing mutated *mca1Ala^426^-X_3_-Ala-Ala-X_3_-Ala^478^-TAP* and *mca1Ala^476^-X-Ala^478^* alleles under copper-limiting and copper-replete conditions.

*A*, *pat1-114*/*pat1-114 mca1Δ*/*mca1Δ* cells containing an empty integrative vector (vector alone) and re-integrated *mca1^+^-TAP*/*mca1^+^-TAP*, *mca1Ala^426^-X_3_-Ala-Ala-X_3_-Ala^478^-TAP* and *mca1Ala^476^-X-Ala^478^* alleles were synchronously induced to undergo meiosis under copper-starved (TTM, 150 μM) and copper-replete (Cu, 25 μM) conditions. Total RNA was isolated from culture aliquots taken at the indicated time points after meiotic induction. Following RNA isolation, *mfc1^+^* and *act1^+^* steady-state mRNA levels were analyzed by RNase protection assays. *B*, Graphic representation of the quantification of the results of three independent RNase protection assays, including assays shown in panel *A*. Values are represented as averages ± S.D. The asterisks correspond to p<0.0001 (^****^) (paired Student’s *t*-test).
